# Cortisol Regulates PD‐1 and IL‐12 in Canine Leishmaniasis

**DOI:** 10.1111/pim.70062

**Published:** 2026-02-02

**Authors:** Lucas Takeshi Siqueira Ito, Gisele Mitsue Umino, Mayla Abbas Guimarães, Bianca Maciel Marques de Souza, Sofia Furrier Soares, Luiz Eduardo Amador Loiola Pereira, Valéria Marçal Felix de Lima

**Affiliations:** ^1^ Department of Clinical Medicine, Surgery and Animal Reproduction São Paulo State University (UNESP) Araçatuba Brazil

**Keywords:** dogs, glucocorticoids, hormonal disorders, visceral leishmaniasis

## Abstract

Canine visceral leishmaniasis (CanL) is a tropical zoonosis caused by Brazil's protozoan *Leishmania* (*L*.) *infantum*. Disorders in the hypothalamic–pituitary–adrenal (HPA) axis have been reported in human and experimental visceral leishmaniasis, but not yet in canine leishmaniasis. Cortisol is a steroid hormone that regulates several processes, including immune responses. This study investigated HPA axis disorders in dogs with visceral leishmaniasis and their link to clinical and immunological parameters. ELISA quantified serum levels of cortisol and adrenocorticotrophic hormone (ACTH) in 12 healthy dogs and 13 dogs with leishmaniasis. The expression of the enzymes inducible nitric oxide synthase (iNOS) and arginase‐1 and programmed cell death protein‐1 (PD‐1) were evaluated by flow cytometry in peripheral blood mononuclear cells (PBMC). Additionally, serum levels of the cytokines interleukin (IL)‐1β, IL‐6, IL‐10, IL‐12, interferon‐gamma (IFN‐γ), tumour necrosis factor‐alpha (TNF‐α), and transforming growth factor‐beta (TGF‐β) were quantified by capture Enzyme‐Linked Immunosorbent Assay (ELISA). Parasite load was quantified in peripheral blood and conjunctival swabs by real‐time polymerase chain reaction (qPCR). All parameters evaluated were correlated with serum cortisol. We observed an increase in cortisol, while ACTH levels were reduced in dogs with leishmaniasis. The expression of iNOS, arginase‐1 and PD‐1 was higher in the PBMC of dogs with leishmaniasis. Serum levels of the cytokines IL‐10, IL‐6, IL‐12, and IFN‐γ were increased in dogs with leishmaniasis. Cortisol showed a negative correlation with PD‐1 and IL‐12. Our findings suggest that infection natural with *L. infantum* in dogs may induce dysregulation of the HPA axis, leading to elevated serum cortisol levels and modulation of the immune response, as it is associated with immunological markers involved in disease pathogenesis. These results contribute to a better understanding of the pathogenic mechanisms of the disease.

## 1 Introduction

Visceral Leishmaniasis (VL) is a zoonosis caused by intracellular hemoflagellate protozoa belonging to the family *Trypanosomatidea* of the genus *Leishmani*a, with *Leishmania* (*L*.) *infantum* (synonymous with *L. chagasi*) being the primary etiologic agent in Brazil [1]. Although estimates for global incidence of VL remain between 50,000 and 90,000 cases, the number of officially notified cases in 2024 was approximately 18,700. This discrepancy reflects the persistent challenge of underreporting and inadequate surveillance in many endemic areas [2]. Brazil is considered an endemic country in the Americas for the disease, concentrating 96% of the cases recorded in the region and 12% of the global burden [2]. Domestic dogs are considered reservoirs of the disease for humans in urban regions [3], and there is an important correlation between the prevalence of the disease in humans and seropositive dogs, especially in endemic areas [4, 5].

The evolution and clinical manifestation of CanL depend on the balance between the host's ability to induce leishmanicidal mechanisms and the parasite's strategies to suppress or evade the host's immune response [6]. The clinical signs are often non‐specific, the most frequent being onychogryphosis, skin lesions, lymphadenopathy, and weight loss [7]. In laboratory tests, some alterations are more frequent, such as anaemia, thrombocytopenia, and hyperglobulinemia associated with hypoalbuminemia, raising total plasma protein [8].

Dogs resistant to infection generally develop a Th1‐type cellular immune response with the production of cytokines, such as IL‐12, which stimulates CD4^+^ T cells to produce two key cytokines, IFN‐γ and TNF‐α [9, 10] activating macrophages to produce nitric oxide (NO) and other reactive oxygen species (hydrogen peroxide and superoxide ion), leading to the intracellular death of the protozoan [11, 12]. Asymptomatic or moderately affected dogs produce high levels of IFN‐γ, whereas reduced IFN‐γ production has been observed in dogs with chronic stages of the disease [13]. Conversely, dogs susceptible to the disease show a Th2‐type humoral response, with exacerbated production of antibodies [13] and production of anti‐inflammatory cytokines such as IL‐4, IL‐10, TGF‐β, increasing the survival of the parasite, leading to the appearance of the symptomatic picture of the disease [10].

Interleukin‐10 is an anti‐inflammatory cytokine induced during infection and acts as a key mediator of cellular immune suppression [14]. Increased IL‐10 production is positively correlated with parasite burden and negatively associated with IFN‐γ levels in PBMCs from dogs naturally infected with visceral leishmaniasis [15]. Moreover, in CanL, TGF‐β contributes to immune evasion by impairing the microbicidal activity of macrophages, thereby reducing their ability to control intracellular parasites [16]. Elevated TGF‐β levels have been reported in hepatic and splenic tissues, as well as in cultures of splenic leukocytes from infected dogs [17]. Thus, elevated levels of pro‐inflammatory cytokines such as IFN‐γ and TNF‐α, together with reduced TGF‐β and IL‐10, represent key immunological markers for vaccine research and for advancing the understanding of disease immunopathogenesis in dogs [18].

Since *Leishmania* spp. necessarily replicate in macrophages, for their survival, they depend on the balance of two inducible enzymes, iNOS and arginase 1 [19]. IFN‐γ increases the expression of iNOS in the macrophage, stimulating NO production and leading to the death of the intracellular parasite [20]. High iNOS expression was observed in macrophages containing few or no *L. infantum* amastigotes in canine samples, whereas macrophages with low enzyme expression harboured a high parasite load [21]. However, the protozoan's modulation of the host's immune response causes the macrophage to polarise toward a profile in which enzyme arginase 1 is induced, producing two compounds: L‐ornithine and urea. L‐ornithine is the primary intracellular source of polyamines, which are necessary for the growth of the intracellular parasite [22, 23].

In CanL, the T cell phenotype plays a decisive role in determining the course of the disease and control of parasite replication [9]. T cell exhaustion has been reported during CanL [24]. Exhaustion of CD4^+^ and CD8^+^ T cells has been shown to be associated with the expression of PD‐1, a co‐inhibitory receptor expressed in the lymphocytes of the spleen and peripheral blood of dogs with VL [25]. In clinically affected dogs, increased PD‐1 expression reduces the proliferative capacity of T cells [24] and impairs the microbicidal activity of the macrophage [26].

The pro‐inflammatory cytokines IL‐1 have increased their production during CanL [27]. IL‐1 signalling through its receptor and the adaptor MyD88 triggers an iNOS‐mediated nitric oxide pathway, an important protective mechanism in infected mice [28]. Consistently, elevated IL‐1 expression was also observed in splenic leukocyte supernatants from dogs with CanL [27]. Moreover, renal IL‐1β activation during CanL amplifies inflammation and contributes to glomerular damage [29].

Interleukin‐6 is also elevated in the serum of dogs during disease progression [30]. This cytokine participates in the acute‐phase response together with IL‐1 [31]. In human visceral leishmaniasis, IL‐6 negatively regulates macrophage microbicidal activity, thereby facilitating parasite proliferation [32]. Elevated IL‐6 levels have been associated with severe clinical outcomes and increased mortality [32].

The host's innate and adaptive immune responses increase pro‐inflammatory cytokines that modulate not only the immune system but also the HPA axis, leading to an increase in cortisol, which has already been observed in VL in hamsters [33], mice [34] and humans [35], and in other infectious diseases such as malaria [36], Chagas disease [37], tuberculosis [38], and viral diseases [39], but not yet studied in dogs with VL.

In infectious diseases, cytokines such as IL‐1, IL‐6, and TNF‐α can act directly on the central nervous system, resulting in the activation of neuroendocrine axes, especially the HPA axis, causing increased production of corticotropin‐releasing hormone (CRH) by the hypothalamus, which induces the pituitary gland to synthesize ACTH which, in turn, stimulates the adrenal glands to produce steroids, glucocorticoids, dehydroepiandrosterone (DHEA) and its sulfate ester (DHEAS) [40, 41]. These hormones can act on the immune response during CanL.

Glucocorticoids (GCs) exert both genomic and non‐genomic effects, modulating transcription factors associated with inflammatory signalling, such as nuclear factor kappa B (NF‐κB) [42]. This modulation results in a reduction in the expression of pro‐inflammatory genes, which, consequently, inhibit the production of pro‐inflammatory cytokines, such as IL‐1, IL‐6, and TNF‐α, as well as those related to the Th1‐type immune response, including IL‐2, IL‐12, and IFN‐γ [43]. At the same time, GCs stimulate the production of anti‐inflammatory cytokines such as IL‐4 and IL‐10, promoting a Th2‐type immune response [40, 43].

Glucocorticoids also attenuate T cell activation by modulating T cell receptor (TCR) signalling in murine hybridomas [44]. In addition, they induce PD‐1 expression in mouse T and natural killer (NK) cells during viral infection [45, 46], presenting antigens and modulating the adaptive response. These hormones can also regulate NO production, reducing the expression of iNOS in murine macrophages in response to inflammatory insults [47, 48], which may benefit the parasite's replication.

Still, as a bidirectional system, GCs can decrease chemotaxis to lymphocytes, monocytes, and granulocytes and promote an imbalance of cellular and humoral response observed in patients with rheumatoid arthritis [40]. In different experimental infection models, an imbalance in the HPA axis prevents the host from mounting an adequate immune response, which can influence susceptibility to the disease [41].

In human patients with VL, of both genders, hormonal disturbances have been reported, but little about their influence on the disease has been elucidated [35]. In male golden hamsters experimentally infected with *L. infantum*, high cortisol levels were observed after infection, and all leukocytes, except monocytes, showed a strong negative correlation with cortisol, while transaminases showed a positive correlation suggesting that cortisol may be involved in the pathogenesis of the disease in the liver associated with experimental VL [33]. Cortisol also correlated positively with pro‐ and anti‐inflammatory immune markers and the enzyme arginase‐1. In contrast, IFN‐γ and the iNOS enzyme showed negative correlations, indicating that cortisol may play a crucial role in regulating the immune response to the parasite [33]. Similarly, in female mice experimentally infected with *L. donovani* , high serum cortisol levels were associated with increased IL‐10, TGF‐β, and parasite load in splenocytes [34]. Therefore, in this study, we investigate changes in the HPA axis in CanL, analysing serum cortisol and ACTH levels and their association with clinical, laboratory, and immunological parameters involved in the progression of the disease.

## 2 Materials and Methods

### 2.1 Ethics Statement

The Ethics Committee approved all the procedures and methods used in the study on the Use of Animals—CEUA of FOA, UNESP, Araçatuba/SP (process number 282‐2023).

### 2.2 Dogs Screening

To select the dogs with leishmaniasis, 13 male dogs between 1 and 8 years of age, of various breeds and weights, from the Zoonosis Control Center of Araçatuba/SP or local guardians were considered, provided they had signed a consent form before the samples were taken. CanL was diagnosed using serological methods: ELISA [49] or immunochromatographic test (Teste Rápido DPP/BioManguinhos, BR) for detection of anti‐*Leishmania* antibodies and confirmed by qPCR molecular method for detection of *Leishmania* spp. DNA in blood samples or conjunctival swabs [50]. All the dogs showed at least three characteristic clinical signs of the disease [7]. Hematologic tests (complete blood count) and serum biochemical tests (albumin, alanine aminotransferase (ALT), aspartate aminotransferase (AST), creatinine, alkaline phosphatase (ALP), globulins, total protein, and urea) to assess the renal and hepatic profile of the group were carried out to determine the clinical stage of these animals. According to the previously proposed staging, only dogs in the moderate stage of the disease were selected [8]. Dogs with elevated creatinine or urea levels were discarded from the study, selecting only those in the moderate stage of the disease, to ensure greater homogeneity of the data and minimise immunological dysfunctions associated with uremia resulting from chronic renal insufficiency [51, 52]. In addition, for all the dogs selected, diagnostics were carried out using the qPCR molecular method to detect the DNA of *Ehrlichia* spp. and *Babesia* spp. in peripheral blood samples at a commercial external laboratory Epigene Diagnósticos (Araçatuba, SP, Brazil) to determine possible co‐infections. Dogs with co‐infection were excluded in this study.

We selected 12 male dogs, between 1 and 5 years old, of various breeds and different weights, from local owners, who signed a consent form before the samples were taken to make up the healthy group. All the dogs had negative ELISA and immunochromatographic tests for CanL and no *Leishmania* spp. DNA in blood and conjunctival swab samples through the detection of *Leishmania* spp. by qPCR. The animals were clinically healthy after a physical examination and showed no alterations in hematologic tests or biochemical profiles consistent with other infections.

Only male dogs were used as inclusion criteria to avoid the interference of hormones acting on cortisol production in the female reproductive cycle.

### 2.3 Sample Collection

To minimise the effect of the circadian cycle, blood samples from all the dogs were collected between 8 and 10 AM. Using disposable hypodermic needles and syringes, whole blood was collected by puncturing the jugular veins of the dogs in both groups. Three millilitres (mL) were placed in plastic tubes containing heparin (Becton‐Dickson, USA) (10 IU/mL of blood) to obtain mononuclear cells. To obtain blood serum, 12 mL were placed in plastic tubes containing clot activator (Vacuplast GmbH, Austria) and centrifuged at 3000 rpm for 5 min and separated for evaluation of biochemical markers and serological tests to detect anti‐leishmania antibodies. Two mL of blood serum were stored at −80°C to measure cytokines and the hormones cortisol and ACTH. Another 1 mL of whole blood was placed in a plastic tube containing K2EDTA (Becton‐Dickson, USA) for the blood count and DNA extraction for qPCR. The blood samples for the laboratory tests were immediately taken to an external laboratory for processing.

Conjunctival samples were collected with a sterile swab from both eyes' upper and lower palpebral conjunctiva, placed in DNA/RNA free microtubes (Eppendorf, USA), and stored at −20°C until DNA extraction.

### 2.4 Immunochromatographic and Serological Diagnosis

The DPP immunochromatographic test was carried out following the manufacturer's instructions. The dogs' serum was analysed by indirect ELISA, using total lysate of *L. chagasi* promastigotes (MHOM/BR00/MER02) as the antigen. The technique to prepare and carry out the ELISA followed the previously described protocol [49].

### 2.5 Determination of Leishmania Species in Dogs With Leishmaniasis

*Leishmania* species was determined using PCR‐RFLP [53], comparing the restriction profile of the sample with the profile obtained from *L. infantum* (IOC/L0575‐MHOM/BR/2002/LPC‐RPV). Water was used as a negative control. A representative figure with 11 samples demonstrated a restriction pattern consistent with *L. infantum* (Figure S1).

### 2.6 PBMC Isolation

PBMCs from both groups were isolated using a gradient of Histopaque 1077 (Sigma, USA) following the manufacturer's recommendations. The isolated cells were then washed three times in phosphate‐buffered saline (PBS) pH 7.2 and resuspended in 1 mL of RPMI 1640 (Sigma, USA) supplemented with 10% inactivated bovine foetal serum (BFS) (Gibco, USA), 0.03% L‐glutamine (Sigma, USA), 100 IU/mL penicillin (Sigma, USA) and 100 mg/mL streptomycin (Sigma, USA).

### 2.7 Quantification of iNOS, Arginase 1, and PD‐1 Using Flow Cytometry

To quantify the expression of iNOS and Arginase 1, PBMCs were fixed with 500 μL of fixation buffer (Invitrogen, CA, USA) and incubated for 10 min at room temperature. The cells were centrifuged at 400 *g* for 5 min and washed two times with permeabilization buffer (Invitrogen, CA, USA). The cells were then resuspended with 50 μL of permeabilization buffer (Invitrogen, CA, USA) and incubated with PE‐conjugated rabbit anti‐mouse iNOS polyclonal antibody (BIORBYT, UK) and FITC‐conjugated rabbit anti‐human arginase 1 polyclonal antibody (BIORBYT, UK) for 60 min at 26°C. The mouse iNOS protein (P29477) blast is similar to the canine iNOS protein (NP_001300777.1) of 80.0%; and the human arginase 1 protein (NP_000036.2) blast is similar to the canine arginase 1 protein (XP_038409331) of 92.24%. The PBMCs were incubated with their respective isotype controls (BIORBYT, UK) for 60 min to avoid non‐specific binding at 26°C. The cells were washed in PBS (pH 7.2) with 1% bovine serum albumin (BSA) and stored at 4°C in the dark until analysis.

To quantify the co‐inhibitory receptor PD‐1, the isolated PBMCs were washed with PBS (pH 7.2) and centrifuged at 400 *g* for 5 min. After centrifugation, they were blocked with 1000 μL of PBS with 10% FBS (Gibco, USA) for 30 min at 26°C and centrifuged again at 400 *g* for 5 min. The cells were then incubated with PE‐conjugated anti‐PD1 monoclonal antibody (anti‐human CD279, Clone MIH4, BD Biosciences, USA) (the PD‐1 protein shows many homologies between canine and human species) [54] and with its control isotype (BD Biosciences, USA), as recommended by the manufacturer. The cells were washed in PBS (pH 7.2) with 1% BSA and stored at 4°C in the dark until analysis.

For analysis of iNOS and Arginase‐1 labelling, monocytes were separated by size (*x*‐axis) and granularity (*y*‐axis) to form gate R1 of PBMCs from healthy dogs and dogs with leishmaniasis (Figure S2A). Fluorescence intensities were assessed using the FL‐2 and FL‐1 detectors, respectively. For PD‐1 labelling analysis, the lymphocytes were separated by size (*x*‐axis) and granularity (*y*‐axis), forming the R2 gate of the PBMCs of healthy dogs and dogs with leishmaniasis (Figure S2B). Fluorescence intensity was assessed using the FL‐2 detector. For all targets, 10,000 events were acquired on an Accuri C5 flow cytometer (BD Biosciences, CA, USA) and analysed using BD Accuri C6 Software, version 1.0.264.21 (BD Biosciences, CA, USA).

### 2.8 Measurement of Cortisol and ACTH by Commercial ELISA Kit

Serum cortisol was measured using a commercial QuicKey Pro Canine Cortisol ELISA kit (MyBioSource, San Diego, USA), following the manufacturer's instructions. Following the manufacturer's instructions, the dogs' serum ACTH was assessed using the commercial kit Canine Adrenocorticotropic Hormone (ACTH) ELISA (MyBioSource, San Diego, USA).

### 2.9 Quantification of the Cytokine IL‐1β, IL‐6, IL‐10, IL‐12, IFN‐y, TNF‐α and TGF‐β by Capture ELISA

Cytokines IL‐1β, IL‐6, IL‐10, IL‐12, IFN‐γ, and TNF‐α were quantified in the serum of dogs from both groups using the DuoSet ELISA Development Systems canine kits (R&D Systems, Minneapolis, MN, USA), following the manufacturer's instructions. TGF‐β quantification in the serum of dogs from both groups was carried out using the Quantikine ELISA Canine TGF‐β1 Immunoassay kit (R&D Systems, Minneapolis, MN, USA), following the manufacturer's instructions.

### 2.10 DNA Extraction and *Leishmania infantum* Parasitic Load Quantification

DNA was extracted from the dogs' blood and conjunctiva samples using the commercial DNeasy kit (Quiagen, Valencia, California, 91355, USA) according to the manufacturer's recommendations. The extracted DNA was analyzed in a spectrophotometer 260/280 (NanoDrop Technologies ND 1000 UV/VIS, USA) to assess the degree of purity and quantification.

The parasite load was quantified by real‐time PCR (qPCR) using primers that amplify a 116 bp fragment of *Leishmania* spp. kinetoplast DNA (5′‐CCTATTTTACACCAACCCCCAGT‐3′ and 5′‐GGGTAGGGGCGTTCTGCGAAA‐3′). For the qPCR reaction, 50 ng of purified genomic DNA, 12.5 μL of SYBR Green JumpStart Taq ReadyMix (Sigma‐Aldrich, St. Louis, MO, USA), 900 nM of primer, and 9.5 μL of ultrapure water were standardised for a final reaction volume of 25 μL. The amplification condition used consisted of an initial heating of 94°C for 3 min, followed by 40 cycles of denaturation (94°C for 40 s), annealing (56°C for 30 s), and extension (72°C for 30 s). At the end of amplification, a dissociation curve of the amplified fragment was determined from 60°C to 95°C with an increase of 0.5°C every 5 s. The quantification of parasite DNA from the samples of dogs with leishmaniasis was determined by comparing it to a standard curve (450 ng/0.006 ng) of *L. infantum* promastigote DNA (MHOM/BR00/MER02) with a serial dilution of factor 5. The standard curve and samples were carried out in duplicates.

### 2.11 Statistical Analysis

Statistical analysis was done using GraphPad Prism V10 software (GraphPad Software Inc., La Jolla, CA, USA). Initially, all variables were tested for normality using the Shapiro–Wilk test. Pearson's correlation test was used to analyse the correlation between the serum cortisol level of dogs with leishmaniasis and the blood count, neutrophils, platelets, renal (creatinine and urea) and hepatic (ALT) biochemical markers, protein count (total protein, albumin and globulins), iNOS and Arginase‐1 enzymes and the cytokines IL‐1β, IL‐6, IL‐10, TNF‐α, TGF‐β, and IFN‐γ. Spearman's correlation test correlated serum cortisol levels with clinical signs and leukogram variables (except neutrophils), liver biochemical markers (AST and FA), PD‐1 molecule, and the IL‐12 cytokine.

Unpaired *t‐*test was used to compare the blood count variables (including neutrophils), serum protein biochemistry and renal biochemistry, cortisol, ACTH, PD‐1, IL‐12, and arginase 1 between the groups. Mann–Whitney test was used for variables that did not follow a normal distribution. This approach was used for leukogram, liver biochemistry, iNOS, and cytokine variables. Significance values were considered when *p* < 0.05 for all the analyses described above.

## 3 Results

### 3.1 Clinical and Laboratory Findings

Dogs with leishmaniasis (*n* = 13) showed at least three characteristic clinical signs of the disease, with onychogryphosis, lymphadenopathy and skin lesions being the most frequent [11/13], followed by periocular lesions [9/13], alopecia and hepatosplenomegaly [6/13]. Other characteristic signs, such as cachexia [3/13], seborrhea [4/13], and ophthalmic disorders [4/13] were observed less frequently in our study. The healthy dogs showed no clinical signs of leishmaniasis or other infections (Table S1). The dogs selected in the leishmaniasis group had serological tests that were reactive for anti‐Leishmania antibodies in the indirect ELISA [49]. Seven dogs were reactive in the rapid DPP and *Leishmania* spp. Eleven blood samples and eight conjunctival swab samples from the dogs with leishmaniasis were detected by qPCR (Table S1). Healthy dogs were negative in all the serological and molecular diagnostic tests for leishmaniasis (Table S1).

In addition to lesions, the haematological findings of dogs with leishmaniasis, the red blood cell count, haemoglobin, haematocrit, and lymphocytes were statistically lower when compared to healthy dogs (Table S2). In the biochemical profile, hypoalbuminemia associated with hyperglobulinemia leading to an increase in total plasma protein was the most frequent finding in dogs with leishmaniasis [12/13] (Table S4). Only two dogs with leishmaniasis showed an increase in ALP (Table S4). The dogs in the control group showed no changes in hematologic or biochemical parameters (Tables S1–S4).

### 3.2 Serum Cortisol Increased, and Serum ACTH Decreased in Dogs With Leishmaniasis

**FIGURE 1:**
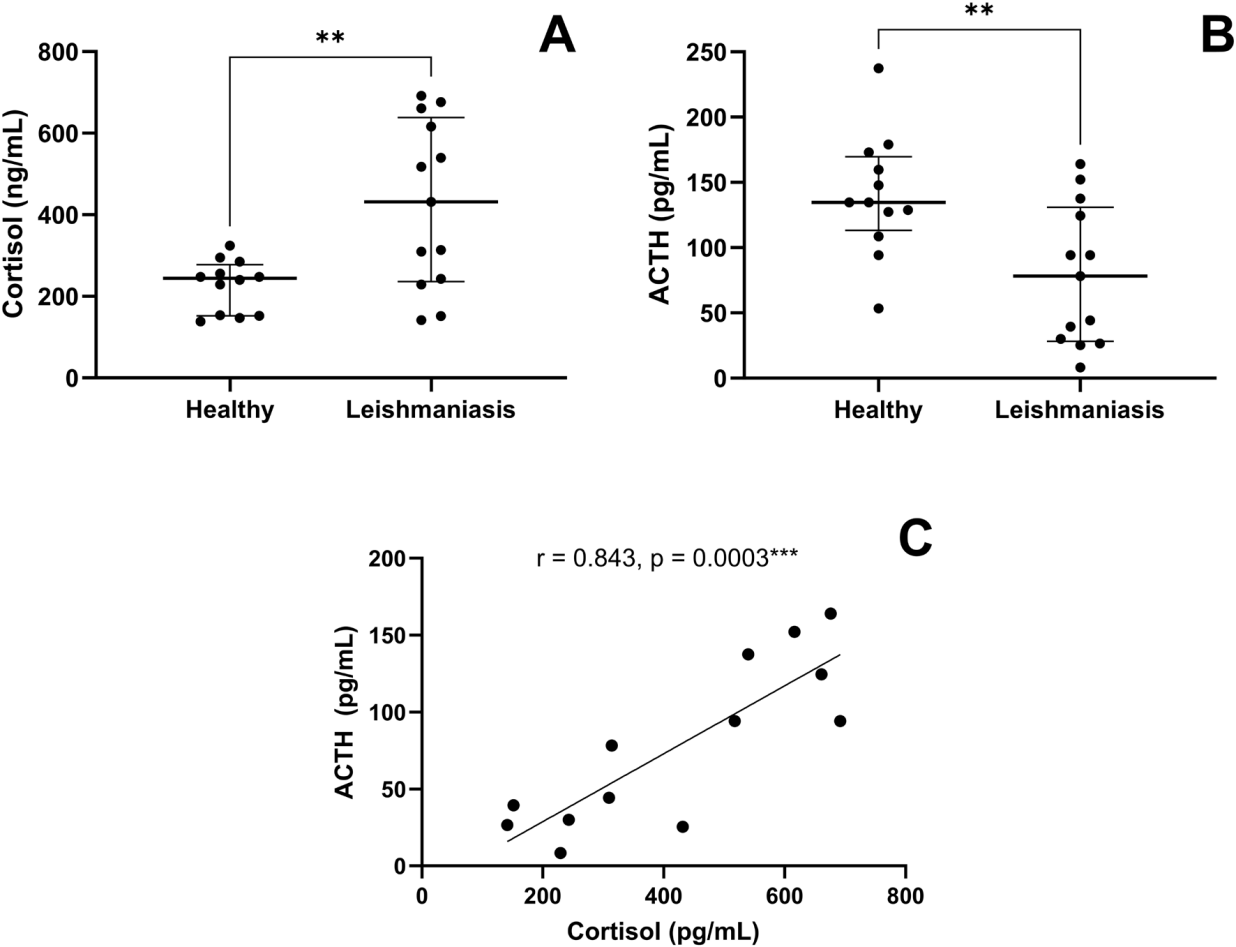
Serum cortisol and ACTH levels in healthy dogs (Healthy) and dogs with leishmaniasis (Leishmaniasis). Correlation serum cortisol and ACTH levels in dogs with leishmaniasis. Serum cortisol (A) and ACTH (B) levels were assessed using the *QuicKey Pro Canine Cortisol* ELISA kit and the commercial *Canine Adrenocorticotropic Hormone* (ACTH) ELISA kit, respectively, in healthy dogs (*n* = 12) and dogs with leishmaniasis (*n* = 13). (C) Correlation between serum cortisol and ACTH levels in dogs with leishmaniasis (*n* = 13) with the fitted linear regression line. Data are expressed as median and interquartile range (25th and 75th percentiles). Symbols represent individual data for each animal. Unpaired *t‐*test was performed to compare the groups. The Pearson's correlation test assessed the association between cortisol and ACTH levels. Asterisks indicate significant differences (“*r*” represents the correlation coefficient) (*p < 0.05, **p < 0.01).

Infectious diseases can lead to hormonal disturbances in the host [41]. Plasma cortisol levels were elevated during infection with *L. infantum* in hamsters [33], mice [34] and humans [35]. Serum cortisol levels were higher (*p* = 0.0038) (Figure 1A), while serum ACTH was lower (*p* = 0.0055) (Figure 1B) in dogs with leishmaniasis than healthy dogs and showed a positive correlation with cortisol (*r* = 0.843, *p* = 0.0003) (Figure 1C).

### 3.3 Cortisol Showed a Positive Correlation With Albumin in Dogs With Leishmaniasis

In hamsters experimentally infected with VL, cortisol showed a negative association with neutrophil, lymphocyte, and monocyte counts and a positive association with the liver biochemical markers ALT, AST, and ALP [33]. We evaluated the correlation between serum cortisol levels and clinical and laboratory parameters in dogs with leishmaniasis. Cortisol showed a positive correlation with serum albumin (Table 1).

**TABLE 1:** Correlations between serum cortisol levels and clinical, serological, hematologic, and biochemical findings in dogs with leishmaniasis.

Variables	Cortisol level's	
	*r* ^a^	*p* ^b^
Clinical signs	−0.370	0.212
O.D. (ELISA)	−0.303	0.313
Red blood cells	0.484	0.093
Haemoglobin	0.519	0.069
Haematocrit	0.489	0.089
MCV	0.123	0.688
MCHC	0.338	0.258
Neutrophils	−0.098	0.747
Lymphocytes	0.098	0.574
Monocytes	0.153	0.616
Eosinophils	0.357	0.231
Platelets	0.369	0.214
**Albumin**	**0.718**	**0.005**
ALT	−0.358	0.228
AST	−0.357	0.229
Creatinine	0.056	0.855
ALP	−0.430	0.142
Globulin	−0.462	0.111
Total protein	−0.104	0.735
Urea	−0.251	0.408

Abbreviations: Mean corpuscular volume (MCV); mean corpuscular haemoglobin concentration (MCHC); optical density (O.D.). Variables in bold indicate significant correlations.

^a^ Correlation assessed using Pearson's test for parametric distributions and Spearman's test for non‐parametric distributions; correlation *r* values represent coefficients; *n* = 13.

^b^ Significance level *p* < 0.05.

### 3.4 iNOS and Arginase 1 Was Elevated in PBMCs From Dogs With Leishmaniasis; However, It Showed No Association With Cortisol

Glucocorticoids can influence macrophage polarisation by negatively regulating transcription factors responsible for increased iNOS expression during inflammatory stimuli [48]. Additionally, in *L. donovani* ‐infected macrophages treated with DHEA, the immunosuppressive activity of cortisol is inhibited, leading to increased NO production [34]. Using flow cytometry, we evaluated the expression of the enzymes iNOS and Arginase‐1 in PBMCs from dogs with leishmaniasis. iNOS is involved in the macrophage production of NO, which mediates microbicidal activity, while Arginase‐1 participates in the synthesis of polyamines, essential for the survival of the intracellular parasite [19].

**FIGURE 2:**
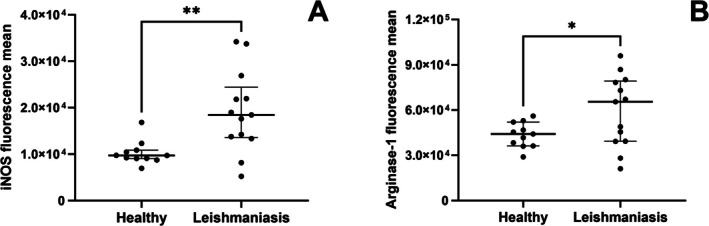
Expression of iNOS and Arginase‐1 enzymes in PBMCs from healthy dogs (Healthy) and dogs with leishmaniasis (Leishmaniasis). Expression levels of iNOS (A) and Arginase‐1 (B) were measured in PBMCs from healthy dogs (healthy group, *n* = 10) and dogs with leishmaniasis (leishmaniasis group, *n* = 13) by flow cytometry. Data are presented as the median and interquartile range (25th and 75th percentiles). Symbols represent individual data points for each animal. Mann–Whitney test was performed for group comparisons for iNOS and unpaired *t*‐test was performed for group comparisons for Arginase‐1. Asterisks indicate significant differences (*p < 0.05, **p < 0.01).

We observed an increase in iNOS and Arginase 1 expression in PBMCs from dogs with leishmaniasis (*p* = 0.0048; *p* = 0.0245) (Figure 2A,B). Representative histograms of fluorescence intensity differences in flow cytometry are shown in Figure S3. Additionally, we observed that Arginase‐1 expression was higher than iNOS in PBMCs from dogs with leishmaniasis (Figure S4). To assess whether cortisol is related to the polarisation of infected macrophages and consequently to parasite survival, we correlated cortisol levels with the expression of iNOS, Arginase‐1, and parasite load. We observed that cortisol showed no correlation with iNOS, Arginase‐1, or blood parasite load during canine leishmaniasis (Table S5).

### 3.5 The PD‐1 Receptor Is Upregulated in PBMCs and Correlates Negatively With Cortisol in Dogs With Leishmaniasis

**FIGURE 3:**
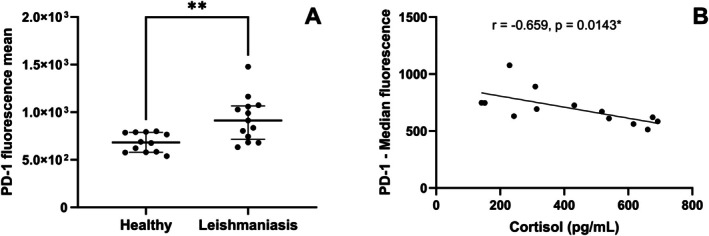
Expression of the co‐inhibitory molecule PD‐1 in PBMCs from healthy dogs (Healthy) and dogs with leishmaniasis (Leishmaniasis). Correlation of PD‐1 with serum cortisol levels in dogs with leishmaniasis. (A) PD‐1 expression in PBMCs from healthy dogs (*n* = 12) and dogs with leishmaniasis (*n* = 13) were analysed by flow cytometry. Data are presented as the median and interquartile range (25th and 75th percentiles). Symbols represent individual data points for each animal. Unpaired *t‐*test was performed for group comparisons. Asterisks indicate significant differences. (B) Correlation between serum cortisol levels and median PD‐1 fluorescence intensity with the fitted linear regression line. Pearson's correlation test assessed the association between cortisol and PD‐1 fluorescence. (“*r*” represents the correlation coefficient; *p < 0,05, **p < 0.01).

PD‐1 plays a crucial role in regulating lymphocyte activation by binding to ligands expressed on antigen‐presenting cells, contributing to maintaining immune tolerance, homeostasis, and prevention of excessive immune responses [24, 25]. The role of PD‐1 during CanL may be associated with the inhibition of T cell activation, thereby regulating and dampening the immune response against the parasite [26]. We observed that PD‐1 was more highly expressed in PBMCs from dogs with leishmaniasis (*p* = 0.0030) (Figure 3A) than in healthy dogs. The representative histogram of fluorescence intensity differences from flow cytometry is shown in Figure S5.

An important action of GCs is the modulation of PD‐1 expression on T lymphocytes and NK cells during cancer [45, 55]. Serum cortisol levels negatively correlated with PD‐1 expression in canine leishmaniasis (*r* = −0.659, *p* = 0.0143) (Figure 3B).

### 3.6 IL‐10, IL‐12, and IFN‐γ Levels Are Elevated in Dogs With Leishmaniasis

Resistance to leishmaniasis has been associated with pro‐inflammatory cytokines such as IL‐1β, IL‐6, IL‐12, TNF‐α, and IFN‐γ [9, 11]. Conversely, susceptibility to the disease is related to the action of immunoregulatory cytokines like TGF‐β and IL‐10 [16, 17]. Therefore, we evaluated the cytokine production in the immune response in dogs with *L. infantum* serum using capture ELISA with specific commercial kits.

**FIGURE 4:**
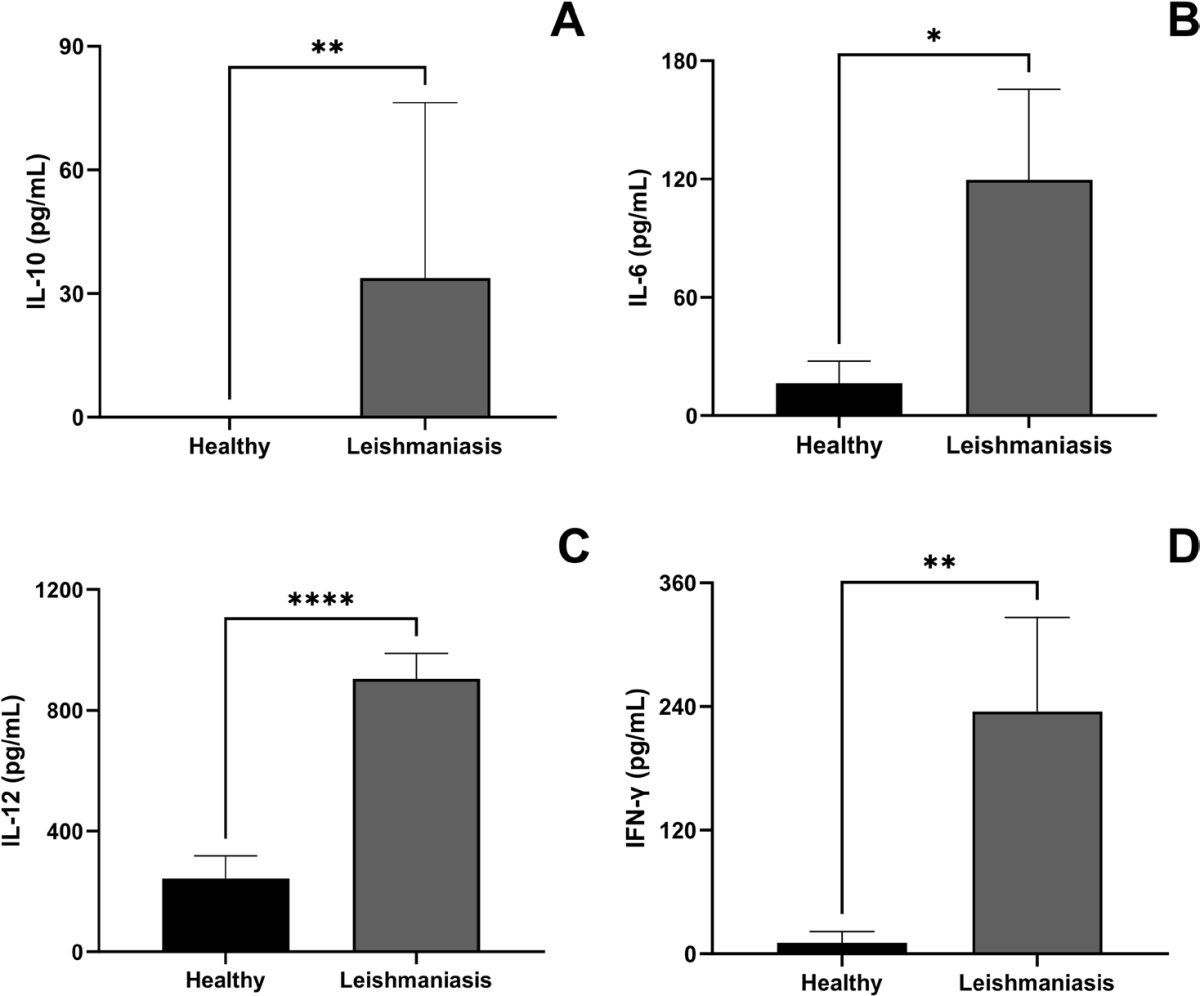
Serum levels of IL‐10, IL‐6, IL‐12 and IFN‐γ in healthy dogs (Healthy) and dogs with leishmaniasis (Leishmaniasis). Serum cytokine levels of IL‐10 (A), IL‐6 (B), IL‐12 (C) and IFN‐γ (D) were measured by capture ELISA using commercial kits in healthy dogs (*n* = 11), dogs with leishmaniasis (*n* = 13). Data are presented as bars representing the mean cytokine levels with the standard error of the mean for each group. Mann–Whitney test was performed for group comparisons for IL‐10, IL‐6 and IFN‐γ and Unpaired *t‐*test was performed for group comparisons for IL‐12. Asterisks indicate significant differences (*p < 0.05, **p < 0.01, ****p < 0.0001).

We observed a significant increase in serum levels of IL‐10 (*p* = 0.005), IL‐6 (*p* = 0.0163), IL‐12 (*p* < 0.0001), and IFN‐γ (*p* = 0.0042) in dogs with leishmaniasis (Figure 4A–D) However, serum levels of IL‐1β, TGF‐β and TNF‐α did not differ significantly between groups (*p* > 0.05) (Figure S6).

### 3.7 Serum Cortisol Showed a Negative Correlation With IL‐12 in Dogs With Leishmaniasis

**FIGURE 5:**
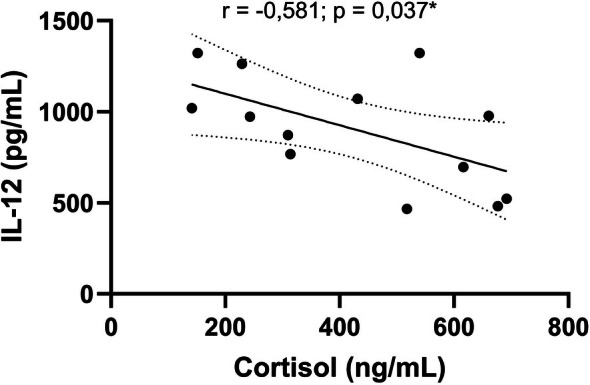
Correlation between serum IL‐12 levels and serum cortisol levels in dogs with leishmaniasis. Correlation between serum cortisol levels and IL‐12 with the fitted linear regression line. Symbols represent individual data points for each animal. Pearson correlation test was performed to assess the association between cortisol and IL‐12 (“*r*” represents the correlation coefficient; *p < 0.05).

Spleen of hamsters with VL showed a positive correlation between cortisol with the mRNA levels of IL‐1β, IL‐6, IL‐10, and TGF‐β and a negative correlation with IFN‐γ [33]. To assess the relationship between cortisol and the immune response in CanL, we performed a correlation analysis between serum cortisol levels and cytokine levels. IL‐1β, IL‐6, IL‐10, and TNF‐α showed no significant correlation with cortisol (Table S5). However, we observed a negative correlation with IL‐12 (*r* = −0.581, *p* = 0.0373) (Figure 5) in dogs with leishmaniasis.

## 4 Discussion

In CanL, parasite survival is associated with an ineffective host response, which induces an anti‐inflammatory profile characterised by an exacerbated humoral response and a weak cellular response [56]. It is known that elevated concentrations of GCs can attenuate the secretion of pro‐inflammatory cytokines and promote a humoral response profile [40, 41]. However, endocrine regulation of the immune response in dogs with leishmaniasis has not yet been fully elucidated. In the present study, we investigated disturbances in the HPA axis by determining serum cortisol and ACTH levels in dogs with leishmaniasis and relating these to clinical and laboratory findings, as well as circulating immunological markers involved in disease progression.

The dogs with serological and molecular diagnoses for leishmaniasis presented onychogryphosis, lymphadenopathy, and skin and periocular lesions as the most frequent clinical findings, compatible with the classic signs expected in CanL [7, 57]. Due to the experimental design of the study, we selected animals at a moderate stage of the disease [8], so dogs without characteristic clinical signs or that showed clinical alterations consistent with other diseases were excluded from the study, aiming to reduce variability within the studied group.

In the moderate stage of the disease, medium to high IgG production is observed [8]. An increase in serum globulin was observed in our study. The high concentration of antibodies favours binding to parasite antigens, leading to the formation of immune complexes deposited in organs such as the kidneys, joints, and eyes [29], a process responsible for the clinical manifestations observed in sick dogs. Changes in renal serum biochemistry were not observed due to the study's inclusion conditions.

During experimental VL in hamsters, GC cortisol appears to be an important link in regulating immunity and disease progression [33] In dogs with leishmaniasis, high serum levels of cortisol, but not ACTH, were observed. Like our findings, human patients with VL and mice infected with *L. donovani* had high levels of cortisol [34, 35]. HPA axis disorders have been observed in other models of systemic infections, such as malaria [36] and Chagas disease [37], suggesting that infection by some protozoa may act by dysregulating the HPA axis, thereby systematically elevating cortisol levels.

It is known that the action of GCs on immune cells occurs mainly through their interaction with their glucocorticoid receptor (GR) with various transcription factors through protein–protein interaction [42]. Among their actions already studied is the inhibition of factors that act in pro‐inflammatory responses and cell differentiation, such as NF‐κB [47], AP‐1 (activator protein 1) [58], T‐Bet (T‐box transcription factor 21) [59], NFAT (nuclear factor of activated T cells) [60] and STAT (signal transducer and activator of transcription) 4 [61]. In contrast, cortisol may favour the activation of STAT3 [62] and STAT5 [63], promoting the expression of genes related to immune regulation and the production of anti‐inflammatory cytokines, inducing a TH2 response profile, directly impacting innate and adaptive immunity during infections. It is possible that during CanL, cortisol aids in the immunosuppression developed in the immunopathogenesis of the disease through cortisol‐GR interaction on immune transcription factors.

Interestingly, we observed a decrease in ACTH in dogs with leishmaniasis. Similarly, ACTH had an initial increase and then a decrease in plasma ACTH concentration in rats experimentally infected with *Trypanosoma brucei* [64]. The regulation of CRH and ACTH is regulated by negative feedback from circulating cortisol, which acts directly on the hypothalamus and pituitary gland [43, 65] through its receptors in the glands [66]. Moreover, external and internal stressors can disrupt the HPA axis by increasing cortisol production [67]. The positive correlation observed between cortisol and ACTH in our dogs suggests that physiological feedback remains preserved. However, the reduction in ACTH combined with elevated cortisol levels may reflect dysregulation of the HPA axis during infection with *L. infantum*.

High iNOS expression is observed in dogs with leishmaniasis. This enzyme is directly associated with NO production, which plays a critical role in the elimination of *Leishmania* within infected macrophages [21]. Elevated iNOS expression has also been observed in other parasitic diseases, including infections caused by *Toxoplasma gondii* [68] and *Neospora caninum* [69]. Collectively, these findings suggest that iNOS expression is a general marker of infection, rather than a disease‐specific indicator.

Cortisol was not associated with the expression of iNOS, arginase‐1, or parasite load in blood monocytes of dogs with leishmaniasis in our study. In contrast, high plasma cortisol levels are negatively associated with iNOS production and positively associated with arginase‐1 production and parasite load in spleen macrophages of hamsters with VL [21]. Similarly, mice experimentally infected with *L. donovani* with high splenic parasite loads also had high cortisol levels, suggesting a regulatory role for the hormone in macrophage microbicidal activity [34]. Thus, a limitation of our research was not evaluating cortisol regulation in the expression of iNOS, arginase‐1, and parasite load in splenic macrophages.

To explore possible cortisol‐regulated immune markers, we observed high expression of the inhibitory molecule PD‐1 in dogs with leishmaniasis. A similar finding was described in splenocytes from dogs with leishmaniasis [24, 70]. Persistent antigenic stimulation drives sustained PD‐1 expression and impairs immune‐mediated clearance of pathogens or dysfunctional cells [71]. Comparable patterns of PD‐1 upregulation have been described in other parasitic and infectious diseases contributing to T cell exhaustion and reduced pathogen control [70, 71, 72]. Although GCs positively modulate PD‐1 expression in T and NK cells in the tumour context [45, 46], during CanL, this association was not observed, suggesting that transcriptional regulatory mechanisms of PD‐1 are distinct in tumours and CanL. Glucocorticoid receptors may increase the binding activity of the transcription factor STAT5 [63]. STAT‐5 competes with NFAT for binding to the *pdcd1* gene, blocking PD‐1 induction in IL‐2‐stimulated T cells [73]. Thus, it is possible that during CanL, endogenous cortisol binds to the GR in T lymphocytes, regulating transcriptional factors such as STAT‐5 involved in the regulation of PD‐1.

Although IL‐10, IL‐6, IL‐12, and IFN‐γ cytokines were elevated in dogs with leishmaniasis, only IL‐12 showed a negative correlation with cortisol. Like this finding, dexamethasone, an exogenous GC, inhibits IL‐12p40 production in LPS‐stimulated human monocytes [74, 75] and alters the response profile from Th1 to Th2 [76] which is beneficial for the survival of *L. infantum*. Interleukin‐12 is essential for the differentiation of TH1 lymphocytes [77]. The IL‐12 p40 subunit gene promoter contains sequences responsive to transcription factors such as activator protein‐1 and NF‐κB in humans and mice [78, 79]. As described above, GCs and their GR can inhibit these factors through protein–protein interaction [32, 37], decreasing IL‐12 production. In addition, T‐bet is also inhibited due to GC binding to its receptor [59] strengthening the mechanisms of cortisol's regulatory role in TH1/TH2 polarization. These findings suggest that cortisol acts on CanL, regulating the immune response in cytokine production that modulates the T cell response profile.

This study shows that high cortisol and low ACTH levels are observed during CanL. Glucocorticoids are generally produced by the adrenal glands through the action of ACTH on its melanocortin 2 receptor (MC2R) [80]. The increase in cortisol in the VL in mice is mediated by the parasite's lipophosphoglycan, which induces the production of prostaglandin E2, which, in its active form 15‐keto‐PGE2, binds to the promoter of 11β‐HSD1 (the enzyme responsible for breaking down cortisone into cortisol), increasing local cortisol production and suggesting a possible evasion mechanism by the parasite [34]. In addition, the presence of the parasite in the pituitary gland [81] and in the adrenal gland [82] in dogs with leishmaniasis reinforces the hypothesis that elevated cortisol is not merely a consequence of infection but a strategy employed by *Leishmania* to modulate the host's immune response.

We conclude that HPA axis dysregulation, characterised by high cortisol levels and low ACTH levels, may play a role in the pathogenesis of CanL due to cortisol's relationship with the expression of co‐stimulatory molecules, such as PD‐1 and with the cytokine IL‐12, which acts on T cell polarisation. Further studies are needed to elucidate better the mechanisms underlying HPA axis dysregulation and other endocrine axes in disease progression and their therapeutic implications from the perspective of immune response and parasite survival.

## Author Contributions

Lucas Takeshi Siqueira Ito performed experiments, acquisition of data, analysis, interpretation, and drafting of the manuscript. Gisele Mitsue Umino, Mayla Abbas Guimarães, Bianca Maciel Marques de Souza, Sofia Furrier Soares, and Luiz Eduardo Amador Loiola Pereira assisted in the experiments and analysed data. Valéria Marçal Felix de Lima was responsible for project guidance, analysis, interpretation, and final drafting of the manuscript. All authors have read and approved the final manuscript.

## Funding

This work was supported by Fundação de Amparo à Pesquisa do Estado de São Paulo, 2023/04301‐7, 2023/04680‐8.

## Conflicts of Interest

The authors declare no conflicts of interest.

## Supporting information


**Figure S1:** RFLP analysis of ITS1‐PCR amplified fragments from DNA samples of standard isolates using the Hae III enzyme. M, molecular marker (100–2000 bp); LI, *Leishmania infantum*. The samples from dogs with leishmaniasis were identical to those of the *L. infantum* sample. RFLPs were identified on 2% agarose gels stained with red gel, as indicated by an arrow.

**Figure S2:** Representative dot plot for gate strategy for selecting monocytes (A) and lymphocytes (B) from a dog with leishmaniasis. Flow cytometry analyses were performed to assess the fluorescence intensity of iNOS, Arginase‐1, and PD‐1 on PBMCs from dogs with leishmaniasis and healthy dogs. Monocytes (A) and lymphocytes (B) were separated by size and granularity to form gates R1 and R2, respectively. Representative dot plots from a dog with leishmaniasis. Dots represent individual cells. Gates selected in red represent the percentage of monocytes and lymphocytes. FSC‐A (forward scatter) and SSC‐A (side scatter).

**Figure S3:** Representative histogram of flow cytometry analysis of iNOS and Arginase 1 production in dogs. iNOS (A) and Arginase‐1 (B) production in PBMCs from dogs were evaluated by flow cytometry and analysed from Gate R1.

**Figure S4:** Expression of iNOS and Arginase‐1 enzymes in PBMCs from dogs with leishmaniasis. iNOS and Arginase‐1 expression was assessed in PBMCs from dogs with leishmaniasis (leishmaniasis group, *n* = 13) and analysed by flow cytometry. Data are expressed as median and interquartile range (25 and 75). Symbols represent individual data for each animal. Unpaired *t‐*test was performed for group comparisons (****p < 0.0001).

**Figure S5:** Histogram representing flow cytometry analysis of PD‐1 expression labelling in dogs. PD‐1 expression in PBMCs from dogs was evaluated by flow cytometry and analysed from Gate R2.

**Figure S6:** Serum levels of IL‐1β, TGF‐beta and TNF‐α in healthy dogs, dogs with leishmaniasis. Serum levels of cytokines IL‐1β (A), TGF‐beta (B), and TNF‐α were assessed using the capture ELISA method with a commercial kit in healthy dogs (healthy group, *n* = 10) and dogs with leishmaniasis (leishmaniasis group, *n* = 13). The data are expressed in bars indicating the mean cytokine levels with standard error of the mean in each group. Unpaired *t‐*test was performed for group comparisons. Asterisks indicate significant differences (*p* < 0.05).

**Table S1:** Clinical signs, serological and molecular diagnoses for canine leishmaniasis, and molecular diagnosis of hemoparasitosis in dogs with leishmaniasis (Leishmaniasis) and healthy dogs (Healthy).
**Table S2:** Red blood cells count in dogs with leishmaniasis (Leishmaniasis) and healthy dogs (Healthy).
**Table S3:** White blood cell and platelet count in dogs with leishmaniasis (Leishmaniasis) and healthy dogs (Healthy).
**Table S4:** Biochemical parameters of dogs with leishmaniasis (Leishmaniasis) and healthy dogs (Healthy).
**Table S5:** Correlations between serum cortisol levels, immunological markers, and parasite load in dogs with leishmaniasis.

## Data Availability

The data that support the findings of this study are available from the corresponding author upon reasonable request.

## References

[1] P. Ready , “Epidemiology of Visceral Leishmaniasis,” Clinical Epidemiology 6 (2014): 147. 24833919 10.2147/CLEP.S44267 PMC4014360

[2] World Health Organization (2025), https://www.who.int/health‐topics/leishmaniasis#tab=tab_1.

[3] J. Alvar , C. Cañavate , R. Molina , J. Moreno , and J. Nieto , Canine Leishmaniasis (Elsevier, 2004), 1–88. 10.1016/S0065-308X(04)57001-X 15504537

[4] R. G. Teixeira‐Neto , E. S. da Silva , R. A. Nascimento , et al., “Canine Visceral Leishmaniasis in an Urban Setting of Southeastern Brazil: An Ecological Study Involving Spatial Analysis,” Parasites & Vectors 7, no. 1 (2014): 485. 25326767 10.1186/s13071-014-0485-7 PMC4209036

[5] D. N. C. C. Costa , P. M. M. B. Bermudi , L. A. C. Rodas , et al., “Human Visceral Leishmaniasis and Relationship With Vector and Canine Control Measures,” Revista de Saúde Pública 52 (2018): 92. 30484481 10.11606/S1518-8787.2018052000381 PMC6280620

[6] S. Buates and G. Matlashewski , “General Suppression of Macrophage Gene Expression During *Leishmania Donovani* Infection,” Journal of Immunology 166, no. 5 (2001): 3416–3422. 10.4049/jimmunol.166.5.3416 11207299

[7] L. Solano‐Gallego , A. Koutinas , G. Miró , et al., “Directions for the Diagnosis, Clinical Staging, Treatment and Prevention of Canine Leishmaniosis,” Veterinary Parasitology 165, no. 1–2 (2009): 1–18. 19559536 10.1016/j.vetpar.2009.05.022

[8] L. Solano‐Gallego , G. Miró , A. Koutinas , et al., “LeishVet Guidelines for the Practical Management of Canine Leishmaniosis,” Parasites & Vectors 4, no. 1 (2011): 86. 21599936 10.1186/1756-3305-4-86 PMC3125381

[9] A. J. Toepp and C. A. Petersen , “The Balancing Act: Immunology of Leishmaniosis,” Research in Veterinary Science 130 (2020): 19–25. 32109759 10.1016/j.rvsc.2020.02.004 PMC7141962

[10] E. Pinelli , R. Killick‐Kendrick , J. Wagenaar , W. Bernadina , G. del Real , and J. Ruitenberg , “Cellular and Humoral Immune Responses in Dogs Experimentally and Naturally Infected With *Leishmania infantum* ,” Infection and Immunity 62, no. 1 (1994): 229–235. 8262632 10.1128/iai.62.1.229-235.1994 PMC186091

[11] P. M. Kaye , M. Svensson , M. Ato , et al., “The Immunopathology of Experimental Visceral Leishmaniasis,” Immunological Reviews 201, no. 1 (2004): 239–253. 15361245 10.1111/j.0105-2896.2004.00188.x

[12] M. A. Panaro , A. Fasanella , S. Lisi , V. Mitolo , A. Andriola , and O. Brandonisio , “Evaluation of Nitric Oxide Production by *Leishmania Infantum‐Infected* Dog Macrophages,” Immunopharmacology and Immunotoxicology 20, no. 1 (1998): 147–158. 9543705 10.3109/08923979809034814

[13] L. Solano‐Gallego , S. Montserrat‐Sangrà , L. Ordeix , and P. Martínez‐Orellana , “ *Leishmania infantum*‐Specific Production of IFN‐γ and IL‐10 in Stimulated Blood From Dogs With Clinical Leishmaniosis,” Parasites & Vectors 9, no. 1 (2016): 317. 27260142 10.1186/s13071-016-1598-y PMC4893235

[14] I. Mesquita , C. Ferreira , A. M. Barbosa , et al., “The Impact of IL‐10 Dynamic Modulation on Host Immune Response Against Visceral Leishmaniasis,” Cytokine 112 (2018): 16–20. 30017388 10.1016/j.cyto.2018.07.001

[15] P. M. Boggiatto , A. E. Ramer‐Tait , K. Metz , et al., “Immunologic Indicators of Clinical Progression During Canine *L Eishmania Infantum* Infection,” Clinical and Vaccine Immunology 17, no. 2 (2010): 267–273. 20032217 10.1128/CVI.00456-09 PMC2815526

[16] K. R. Gantt , S. Schultz‐Cherry , N. Rodriguez , et al., “Activation of TGF‐β by *Leishmania chagasi*: Importance for Parasite Survival in Macrophages,” Journal of Immunology 170, no. 5 (2003): 2613–2620. 10.4049/jimmunol.170.5.2613 12594289

[17] A. P. F. L. Corrêa , A. C. S. Dossi , R. de Oliveira Vasconcelos , D. P. Munari , and V. M. F. de Lima , “Evaluation of Transformation Growth Factor β1, Interleukin‐10, and Interferon‐γ in Male Symptomatic and Asymptomatic Dogs Naturally Infected by *Leishmania (Leishmania) chagasi* ,” Veterinary Parasitology 143, no. 3–4 (2007): 267–274. 16979825 10.1016/j.vetpar.2006.08.023

[18] R. C. Giunchetti , P. Silveira , L. A. Resende , et al., “Canine Visceral Leishmaniasis Biomarkers and Their Employment in Vaccines,” Veterinary Parasitology 271 (2019): 87–97. 31303211 10.1016/j.vetpar.2019.05.006

[19] T. Naderer and M. J. McConville , “The Leishmania–Macrophage Interaction: A Metabolic Perspective,” Cellular Microbiology 10, no. 2 (2007): 301–308. 18070117 10.1111/j.1462-5822.2007.01096.x

[20] R. Olekhnovitch and P. Bousso , “Induction, Propagation, and Activity of Host Nitric Oxide: Lessons From Leishmania Infection,” Trends in Parasitology 31, no. 12 (2015): 653–664. 26440786 10.1016/j.pt.2015.08.001

[21] R. Zafra , J. R. Jaber , R. A. Pérez‐Écija , A. Barragán , A. Martínez‐Moreno , and J. Pérez , “High iNOS Expression in Macrophages in Canine Leishmaniasis Is Associated With Low Intracellular Parasite Burden,” Veterinary Immunology and Immunopathology 123, no. 3–4 (2008): 353–359. 18406470 10.1016/j.vetimm.2008.02.022

[22] A. Badirzadeh , T. Taheri , Y. Taslimi , et al., “Arginase Activity in Pathogenic and Non‐Pathogenic Species of Leishmania Parasites,” PLoS Neglected Tropical Diseases 11, no. 7 (2017): e0005774. 28708893 10.1371/journal.pntd.0005774 PMC5529023

[23] G. Pessenda and J. S. da Silva , “Arginase and Its Mechanisms in *Leishmania* Persistence,” Parasite Immunology 42, no. 7 (2020): e12722. 32294247 10.1111/pim.12722

[24] K. J. Esch , R. Juelsgaard , P. A. Martinez , D. E. Jones , and C. A. Petersen , “Programmed Death 1‐Mediated T Cell Exhaustion During Visceral Leishmaniasis Impairs Phagocyte Function,” Journal of Immunology 191, no. 11 (2013): 5542–5550. 10.4049/jimmunol.1301810 PMC3896087 24154626

[25] V. M. Chiku , K. L. O. Silva , B. F. M. de Almeida , et al., “PD‐1 Function in Apoptosis of T Lymphocytes in Canine Visceral Leishmaniasis,” Immunobiology 221, no. 8 (2016): 879–888. 27016050 10.1016/j.imbio.2016.03.007

[26] G. T. Rebech , G. L. Venturin , L. T. Siqueira Ito , et al., “PD‐1 Regulates Leishmanicidal Activity and IL‐17 in Dogs With Leishmaniasis,” Veterinary Immunology and Immunopathology 219 (2020): 109970. 31733502 10.1016/j.vetimm.2019.109970

[27] G. T. Rebech , J. P. Bragato , S. F. Costa , et al., “miR‐148a Regulation Interferes in Inflammatory Cytokine and Parasitic Load in Canine Leishmaniasis,” PLoS Neglected Tropical Diseases 17, no. 1 (2023): e0011039. 36719867 10.1371/journal.pntd.0011039 PMC9888699

[28] D. S. Lima‐Junior , D. L. Costa , V. Carregaro , et al., “Inflammasome‐Derived IL‐1β Production Induces Nitric Oxide–Mediated Resistance to Leishmania,” Nature Medicine 19, no. 7 (2013): 909–915. 10.1038/nm.3221 23749230

[29] K. J. Esch , R. G. Schaut , I. M. Lamb , et al., “Activation of Autophagy and Nucleotide‐Binding Domain Leucine‐Rich Repeat‐Containing‐Like Receptor Family, Pyrin Domain‐Containing 3 Inflammasome During Leishmania Infantum–Associated Glomerulonephritis,” American Journal of Pathology 185, no. 8 (2015): 2105–2117. 26079813 10.1016/j.ajpath.2015.04.017 PMC4530124

[30] V. M. F. de Lima , J. R. Peiro , and R. de Oliveira Vasconcelos , “IL‐6 and TNF‐α Production During Active Canine Visceral Leishmaniasis,” Veterinary Immunology and Immunopathology 115, no. 1–2 (2007): 189–193. 17097150 10.1016/j.vetimm.2006.10.003

[31] D. L. Costa , R. L. Rocha , R. M. A. Carvalho , et al., “Serum Cytokines Associated With Severity and Complications of Kala‐Azar,” Pathogens and Global Health 107, no. 2 (2013): 78–87. 23683334 10.1179/2047773213Y.0000000078 PMC4001482

[32] P. L. dos Santos , F. A. de Oliveira , M. L. B. Santos , et al., “The Severity of Visceral Leishmaniasis Correlates With Elevated Levels of Serum IL‐6, IL‐27 and sCD14,” PLoS Neglected Tropical Diseases 10, no. 1 (2016): e0004375. 26814478 10.1371/journal.pntd.0004375 PMC4729473

[33] T. d. D. Barros‐Gonçalves , A. F. Saavedra , L. da Silva‐Couto , et al., “Increased Levels of Cortisol Are Associated With the Severity of Experimental Visceral Leishmaniasis in a *Leishmania* (*L*.) *infantum*‐Hamster Model,” PLoS Neglected Tropical Diseases 15, no. 11 (2021): e0009987. 34813597 10.1371/journal.pntd.0009987 PMC8651114

[34] A. Seth , M. Dutta , R. Sarkar , P. Prusti , S. Katiyar , and S. Kar , “DHEA Counteracts *Leishmania donovani*‐Induced Cortisol: GR Signalling‐Mediated Immunosuppression and Anti‐Inflammatory Bias,” Journal of Infectious Diseases 232 (2025): 1389. 40293810 10.1093/infdis/jiaf223

[35] F. A. Lima Verde , F. A. A. Lima Verde , A. Saboia Neto , P. C. Almeida , and E. M. Lima Verde , “Hormonal disturbances in visceral leishmaniasis (kala‐azar),” American Journal of Tropical Medicine and Hygiene 84, no. 5 (2011): 668–673. 21540373 10.4269/ajtmh.2011.09-0171 PMC3083731

[36] F. Esfandiari , B. Sarkari , H. Turki , N. Arefkhah , and N. Shakouri , “Level of Circulating Steroid Hormones in Malaria and Cutaneous Leishmaniasis: A Case Control Study,” Journal of Parasitic Diseases 43, no. 1 (2019): 54–58. 30956446 10.1007/s12639-018-1055-2 PMC6423213

[37] W. Savino , Endocrine Immunology of Chagas Disease (S. Karger AG, 2017), 160–175. 10.1159/000452914 28245460

[38] A. del Rey , C. V. Mahuad , V. V. Bozza , et al., “Endocrine and Cytokine Responses in Humans With Pulmonary Tuberculosis,” Brain, Behavior, and Immunity 21, no. 2 (2007): 171–179. 16890403 10.1016/j.bbi.2006.06.005

[39] M. N. Silverman , B. D. Pearce , C. A. Biron , and A. H. Miller , “Immune Modulation of the Hypothalamic‐Pituitary‐Adrenal (HPA) Axis During Viral Infection,” Viral Immunology 18, no. 1 (2005): 41–78. 15802953 10.1089/vim.2005.18.41 PMC1224723

[40] J. J. Haddad , N. E. Saadé , and B. Safieh‐Garabedian , “Cytokines and Neuro–Immune–Endocrine Interactions: A Role for the Hypothalamic–Pituitary–Adrenal Revolving Axis,” Journal of Neuroimmunology 133, no. 1–2 (2002): 1–19. 12446003 10.1016/s0165-5728(02)00357-0

[41] A. R. Pérez , O. Bottasso , and W. Savino , “The Impact of Infectious Diseases Upon Neuroendocrine Circuits,” Neuroimmunomodulation 16, no. 2 (2009): 96–105. 19212129 10.1159/000180264

[42] D. W. Cain and J. A. Cidlowski , “Immune Regulation by Glucocorticoids,” Nature Reviews. Immunology 17, no. 4 (2017): 233–247. 10.1038/nri.2017.1 PMC9761406 28192415

[43] S. Miyake , “Mind Over Cytokines: Crosstalk and Regulation Between the Neuroendocrine and Immune Systems,” Clinical and Experimental Neuroimmunology 3, no. 1 (2012): 1–15.

[44] F. Van Laethem , E. Baus , L. A. Smyth , et al., “Glucocorticoids Attenuate T Cell Receptor Signaling,” Journal of Experimental Medicine 193, no. 7 (2001): 803–814. 11283153 10.1084/jem.193.7.803 PMC2193373

[45] N. Maeda , T. Maruhashi , D. Sugiura , K. Shimizu , O. Imi , and T. Okazaki , “Glucocorticoids Potentiate the Inhibitory Capacity of Programmed Cell Death 1 by Up‐Regulating Its Expression on T Cells,” Journal of Biological Chemistry 294, no. 52 (2019): 19896–19906. 31723031 10.1074/jbc.RA119.010379 PMC6937557

[46] L. Quatrini , E. Wieduwild , B. Escaliere , et al., “Endogenous Glucocorticoids Control Host Resistance to Viral Infection Through the Tissue‐Specific Regulation of PD‐1 Expression on NK Cells,” Nature Immunology 19, no. 9 (2018): 954–962. 30127438 10.1038/s41590-018-0185-0 PMC6138242

[47] J. Dong , J. Li , L. Cui , et al., “Cortisol Modulates Inflammatory Responses in LPS‐Stimulated RAW264.7 Cells via the NF‐κB and MAPK Pathways,” BMC Veterinary Research 14, no. 1 (2018): 30. 29378573 10.1186/s12917-018-1360-0 PMC5789647

[48] M. Hämäläinen , R. Lilja , H. Kankaanranta , and E. Moilanen , “Inhibition of iNOS Expression and NO Production by Anti‐Inflammatory Steroids,” Pulmonary Pharmacology & Therapeutics 21, no. 2 (2008): 331–339. 17913526 10.1016/j.pupt.2007.08.003

[49] V. M. F. de Lima , L. Biazzono , A. C. Silva , A. P. F. L. Correa , and M. C. R. Luvizotto , “Serological Diagnosis of Visceral Leishmaniasis by an Enzyme Immunoassay Using Protein A in Naturally Infected Dogs,” Pesquisa Veterinaria Brasileira 25, no. 4 (2005): 215–218.

[50] M. A. Fernandes , J. A. F. Leonel , J. A. Isaac , et al., “Molecular Detection of Leishmania Infantum DNA According to Clinical Stages of Leishmaniasis in Dog,” Revista Brasileira de Parasitologia Veterinária 28, no. 2 (2019): 194–202. 31188942 10.1590/S1984-29612019015

[51] M. Espi , L. Koppe , D. Fouque , and O. Thaunat , “Chronic Kidney Disease‐Associated Immune Dysfunctions: Impact of Protein‐Bound Uremic Retention Solutes on Immune Cells,” Toxins (Basel) 12, no. 5 (2020): 300. 32384617 10.3390/toxins12050300 PMC7291164

[52] M. Girndt , M. Sester , U. Sester , H. Kaul , and H. Köhler , “Molecular Aspects of T‐ and B‐Cell Function in Uremia,” Kidney International 59 (2001): S206–S211. 10.1046/j.1523-1755.2001.59780206.x 11169012

[53] L. d. C. Sanches , C. C. de Martini , A. A. Nakamura , M. E. B. Santiago , B. de Dolabela Lima , and V. M. F. de Lima , “Natural Canine Infection by Leishmania Infantum and Leishmania Amazonensis and Their Implications for Disease Control,” Revista Brasileira de Parasitologia Veterinária 25, no. 4 (2016): 465–469. 27925065 10.1590/S1984-29612016071

[54] A. Folkl , X. Wen , E. Kuczynski , M. E. Clark , and D. Bienzle , “Feline Programmed Death and Its Ligand: Characterization and Changes With Feline Immunodeficiency Virus Infection,” Veterinary Immunology and Immunopathology 134, no. 1–2 (2010): 107–114. 19931185 10.1016/j.vetimm.2009.10.019

[55] S. Adorisio , L. Cannarile , D. V. Delfino , and E. Ayroldi , “Glucocorticoid and PD‐1 Cross‐Talk: Does the Immune System Become Confused?,” Cells 10, no. 9 (2021): 2333. 34571982 10.3390/cells10092333 PMC8468592

[56] L. M. Melo , J. P. Bragato , G. L. Venturin , et al., “Induction of miR 21 Impairs the Anti‐Leishmania Response Through Inhibition of IL‐12 in Canine Splenic Leukocytes,” PLoS One 14, no. 12 (2019): e0226192. 31825987 10.1371/journal.pone.0226192 PMC6905561

[57] R. B. P. Torrecilha , Y. T. Utsunomiya , A. M. Bosco , et al., “Correlations Between Peripheral Parasite Load and Common Clinical and Laboratory Alterations in Dogs With Visceral Leishmaniasis,” Preventive Veterinary Medicine 132 (2016): 83–87. 27664450 10.1016/j.prevetmed.2016.08.006

[58] C. Jonat , H. J. Rahmsdorf , K. K. Park , et al., “Antitumor Promotion and Antiinflammation: Down‐Modulation of AP‐1 (Fos/Jun) Activity by Glucocorticoid Hormone,” Cell 62, no. 6 (1990): 1189–1204. 2169351 10.1016/0092-8674(90)90395-u

[59] A. C. Liberman , D. Refojo , J. Druker , et al., “The Activated Glucocorticoid Receptor Inhibits the Transcription Factor T‐Bet by Direct Protein–Protein Interaction,” FASEB Journal 21, no. 4 (2007): 1177–1188. 17215482 10.1096/fj.06-7452com

[60] D. C. Tsitoura and P. B. Rothman , “Enhancement of MEK/ERK Signaling Promotes Glucocorticoid Resistance in CD4+ T Cells,” Journal of Clinical Investigation 113, no. 4 (2004): 619–627. 14966571 10.1172/JCI18975 PMC338260

[61] A. J. Fahey , R. A. Robins , K. B. Kindle , D. M. Heery , and C. S. Constantinescu , “Effects of Glucocorticoids on STAT4 Activation in Human T Cells Are Stimulus‐Dependent,” Journal of Leukocyte Biology 80, no. 1 (2006): 133–144. 16670125 10.1189/jlb.0605296

[62] Z. Zhang , S. Jones , J. S. Hagood , N. L. Fuentes , and G. M. Fuller , “STAT3 Acts as a co‐Activator of Glucocorticoid Receptor Signaling,” Journal of Biological Chemistry 272, no. 49 (1997): 30607–30610. 9388192 10.1074/jbc.272.49.30607

[63] E. Stöcklin , M. Wissler , F. Gouilleux , and B. Groner , “Functional Interactions Between Stat5 and the Glucocorticoid Receptor,” Nature 383, no. 6602 (1996): 726–728. 8878484 10.1038/383726a0

[64] C. I. Maina , “Plasma ACTH Concentration and Pituitary Gland Histo‐Pathology in Rats Infected With *Trypanosoma brucei brucei* ,” African Health Sciences 17, no. 4 (2018): 1029. 10.4314/ahs.v17i4.10 PMC5870269 29937873

[65] J. S. Cowan and R. A. Layberry , “Feedback Suppression of ACTH Secretion by Cortisol in Dogs: Lags After Large Signals Equal Those Following Very Small Signals,” Canadian Journal of Physiology and Pharmacology 61, no. 11 (1983): 1281–1288. 6318936 10.1139/y83-185

[66] F. Spiga , L. R. Harrison , S. A. Wood , et al., “Effect of the Glucocorticoid Receptor Antagonist Org 34850 on Basal and Stress‐Induced Corticosterone Secretion,” Journal of Neuroendocrinology 19, no. 11 (2007): 891–900. 17927667 10.1111/j.1365-2826.2007.01605.x

[67] R. M. Sapolsky , L. M. Romero , and A. U. Munck , “How Do Glucocorticoids Influence Stress Responses? Integrating Permissive, Suppressive, Stimulatory, and Preparative Actions*,” Endocrine Reviews 21, no. 1 (2000): 55–89. 10696570 10.1210/edrv.21.1.0389

[68] Z. Li , Z. J. Zhao , X. Q. Zhu , et al., “Differences in iNOS and Arginase Expression and Activity in the Macrophages of Rats Are Responsible for the Resistance Against *T. gondii* Infection,” PLoS One 7, no. 4 (2012): e35834. 22558235 10.1371/journal.pone.0035834 PMC3338469

[69] P. d. S. C. Barros , C. M. Mota , V. d. S. Miranda , et al., “Inducible Nitric Oxide Synthase Is Required for Parasite Restriction and Inflammatory Modulation During Neospora Caninum Infection,” Veterinary Parasitology 276 (2019): 108990. 31775103 10.1016/j.vetpar.2019.108990

[70] K. L. Oliveira Silva , V. Marin Chiku , G. Luvizotto Venturin , et al., “PD‐1 and PD‐L1 Regulate Cellular Immunity in Canine Visceral Leishmaniasis,” Comparative Immunology, Microbiology and Infectious Diseases 62 (2019): 76–87. 30711051 10.1016/j.cimid.2018.12.002

[71] J. M. Jubel , Z. R. Barbati , C. Burger , D. C. Wirtz , and F. A. Schildberg , “The Role of PD‐1 in Acute and Chronic Infection,” Frontiers in Immunology 11 (2020): 487. 32265932 10.3389/fimmu.2020.00487 PMC7105608

[72] D. L. Barber , K. D. Mayer‐Barber , C. G. Feng , A. H. Sharpe , and A. Sher , “CD4 T Cells Promote Rather Than Control Tuberculosis in the Absence of PD‐1‐Mediated Inhibition,” Journal of Immunology 186, no. 3 (2011): 1598–1607. 10.4049/jimmunol.1003304 PMC4059388 21172867

[73] G. Wang , M. Tajima , T. Honjo , and A. Ohta , “STAT5 Interferes With PD‐1 Transcriptional Activation and Affects CD8+ T‐Cell Sensitivity to PD‐1‐Dependent Immunoregulation,” International Immunology 33, no. 11 (2021): 563–572. 34453440 10.1093/intimm/dxab059

[74] W. Ma , K. Gee , W. Lim , et al., “Dexamethasone Inhibits IL‐12p40 Production in Lipopolysaccharide‐Stimulated Human Monocytic Cells by Down‐Regulating the Activity of c‐Jun N‐Terminal Kinase, the Activation Protein‐1, and NF‐κB Transcription Factors,” Journal of Immunology 172, no. 1 (2004): 318–330. 10.4049/jimmunol.172.1.318 14688340

[75] M. Mirani , I. Elenkov , S. Volpi , N. Hiroi , G. P. Chrousos , and T. Kino , “HIV‐1 Protein Vpr Suppresses IL‐12 Production From Human Monocytes by Enhancing Glucocorticoid Action: Potential Implications of Vpr Coactivator Activity for the Innate and Cellular Immunity Deficits Observed in HIV‐1 Infection,” Journal of Immunology 169, no. 11 (2002): 6361–6368. 10.4049/jimmunol.169.11.6361 12444143

[76] S. K. Agarwal and G. D. Marshall , “Dexamethasone Promotes Type 2 Cytokine Production Primarily Through Inhibition of Type 1 Cytokines,” Journal of Interferon & Cytokine Research 21, no. 3 (2001): 147–155. 11331037 10.1089/107999001750133159

[77] C. L. Barbiéri , “Immunology of Canine Leishmaniasis,” Parasite Immunology 28, no. 7 (2006): 329–337. 16842269 10.1111/j.1365-3024.2006.00840.x

[78] C. Zhu , K. Gagnidze , J. H. M. Gemberling , and S. E. Plevy , “Characterization of an Activation Protein‐1‐Binding Site in the Murine Interleukin‐12 p40 Promoter,” Journal of Biological Chemistry 276, no. 21 (2001): 18519–18528. 11279072 10.1074/jbc.M100440200

[79] T. L. Murphy , M. G. Cleveland , P. Kulesza , J. Magram , and K. M. Murphy , “Regulation of Interleukin 12 p40 Expression Through an NF‐κB Half‐Site,” Molecular and Cellular Biology 15, no. 10 (1995): 5258–5267. 7565674 10.1128/mcb.15.10.5258 PMC230773

[80] J. P. Herman , J. M. McKlveen , S. Ghosal , et al., “Regulation of the Hypothalamic‐Pituitary‐Adrenocortical Stress Response,” in Comprehensive Physiology (Wiley, 2016), 603–621. 10.1002/cphy.c150015 PMC4867107 27065163

[81] E. D. Frigerio , C. d. C. Guizelini , G. G. Jussiani , et al., “Lymphocytic Hypophysitis in Dogs Infected With Leishmania spp,” Frontiers in Veterinary Science 10 (2023): 1208919. 37781278 10.3389/fvets.2023.1208919 PMC10537919

[82] C. Momo , N. A. d. S. Rocha , P. R. R. Moreira , et al., “Morphological Changes and Parasite Load of the Adrenal From Dogs With Visceral Leishmaniasis,” Revista Brasileira de Parasitologia Veterinária 23, no. 1 (2014): 30–35. 24728358 10.1590/s1984-29612014004

