# Supplementary figures and images for: Cortisol Regulates PD‐1 and IL‐12 in Canine Leishmaniasis
Source: Parasite Immunol. 2026 Feb 2;48(2):e70062. doi: 10.1111/pim.70062 (PMC12862539; doi:10.1111/pim.70062)

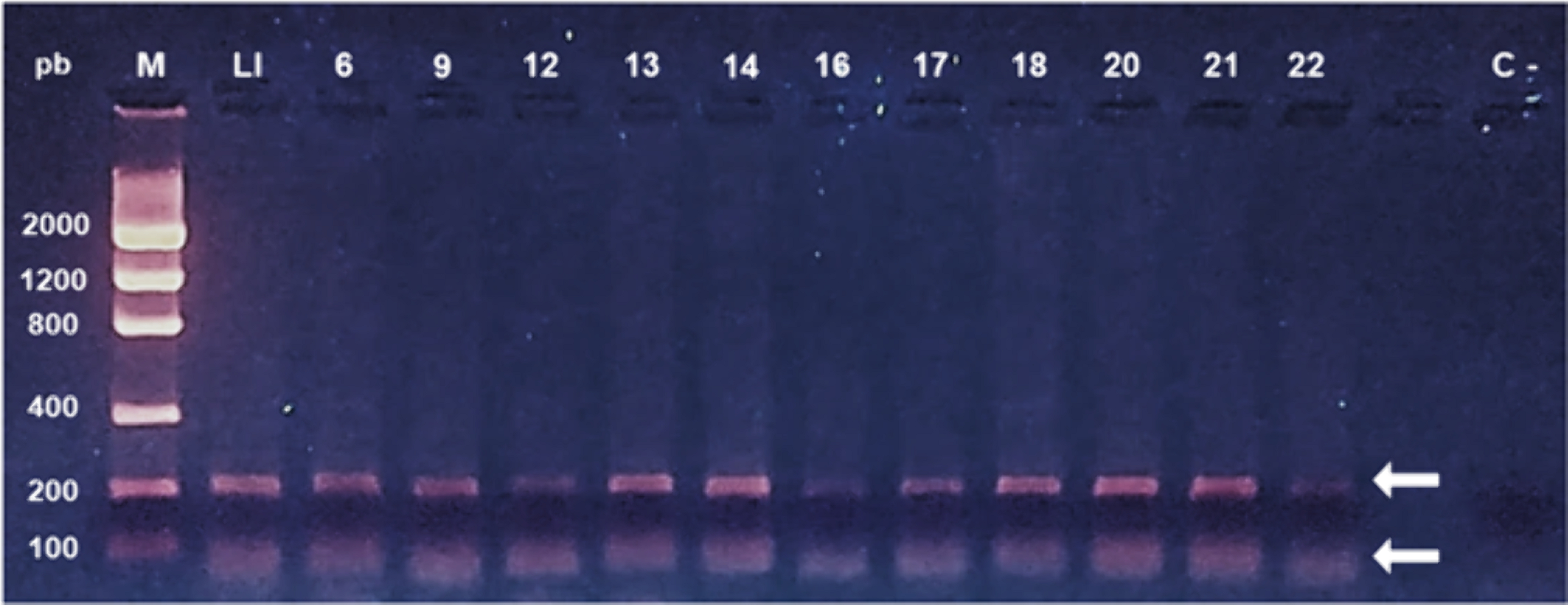

Supplement: Supplementary file 1 — Figure S1: RFLP analysis of ITS1‐PCR amplified fragments from DNA samples of standard isolates using the Hae III enzyme. M, molecular marker (100–2000 bp); LI, Leishmania infantum. The samples from dogs with leishmaniasis were identical to those of the L. infantum sample. RFLPs were identified on 2% agarose gels stained with red gel, as indicated by an arrow. [file PIM-48-e70062-s005.tif]

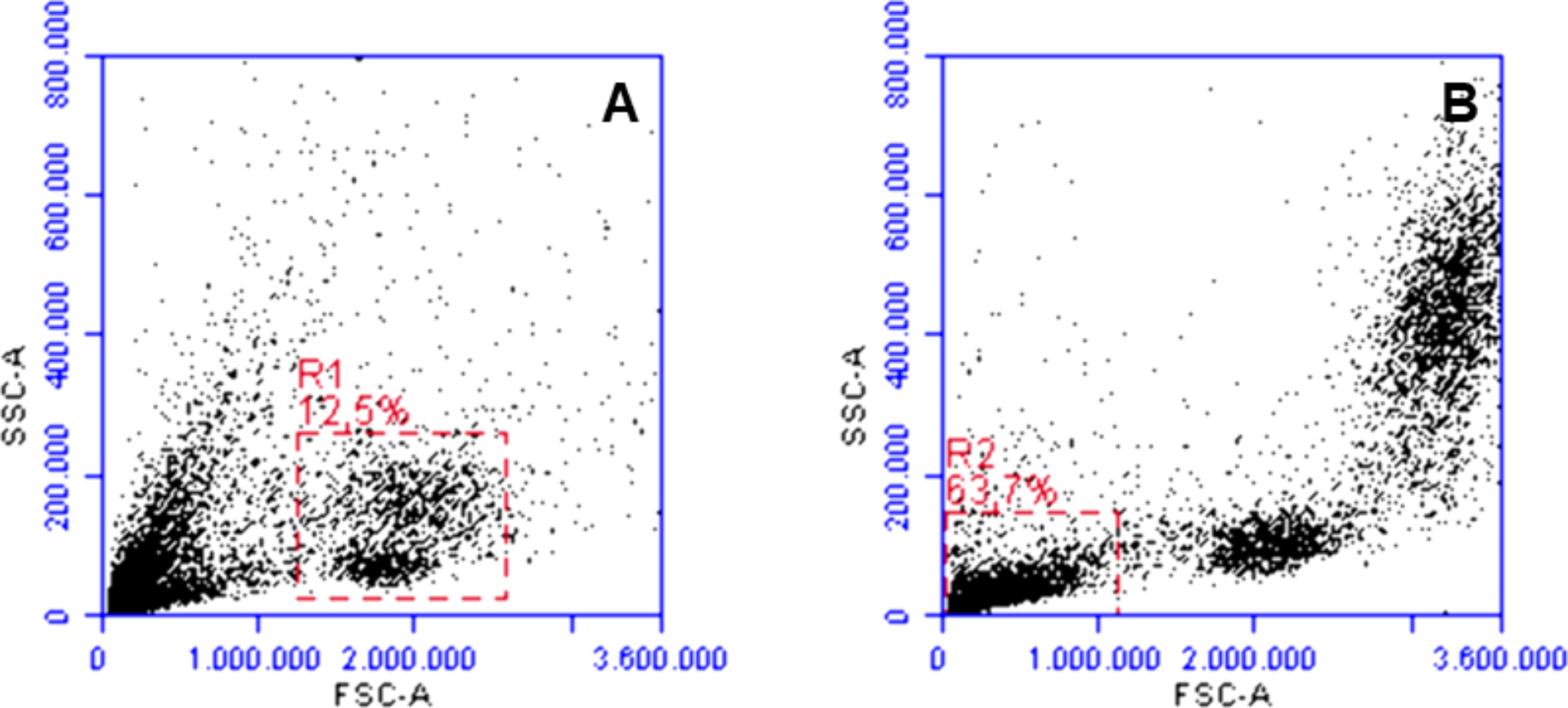

Supplement: Supplementary file 2 — Figure S2: Representative dot plot for gate strategy for selecting monocytes (A) and lymphocytes (B) from a dog with leishmaniasis. Flow cytometry analyses were performed to assess the fluorescence intensity of iNOS, Arginase‐1, and PD‐1 on PBMCs from dogs with leishmaniasis and healthy dogs. Monocytes (A) and lymphocytes (B) were separated by size and granularity to form gates R1 and R2, respectively. Representative dot plots from a dog with leishmaniasis. Dots represent individual cells. Gates selected in red represent the percentage of monocytes and lymphocytes. FSC‐A (forward scatter) and SSC‐A (side scatter). [file PIM-48-e70062-s002.tiff]

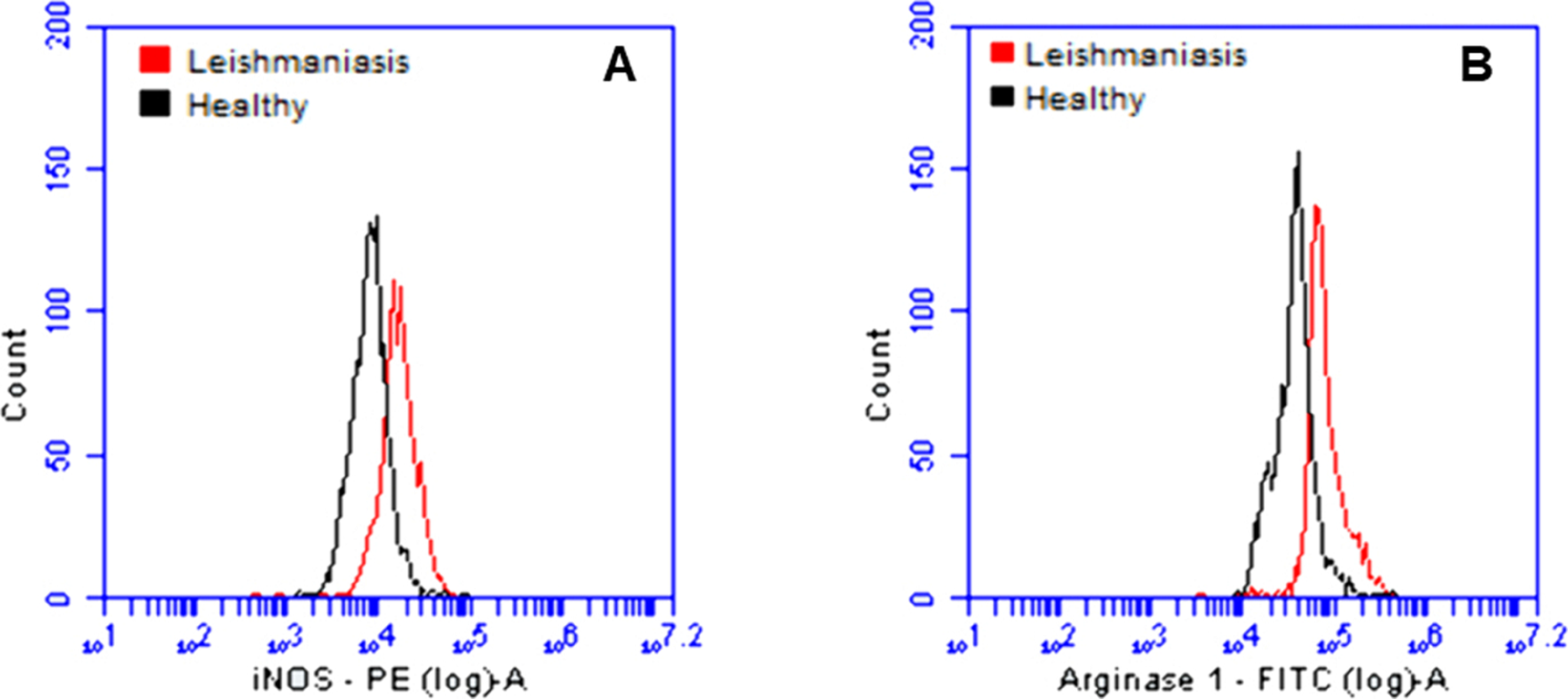

Supplement: Supplementary file 3 — Figure S3: Representative histogram of flow cytometry analysis of iNOS and Arginase 1 production in dogs. iNOS (A) and Arginase‐1 (B) production in PBMCs from dogs were evaluated by flow cytometry and analysed from Gate R1. [file PIM-48-e70062-s003.tif]

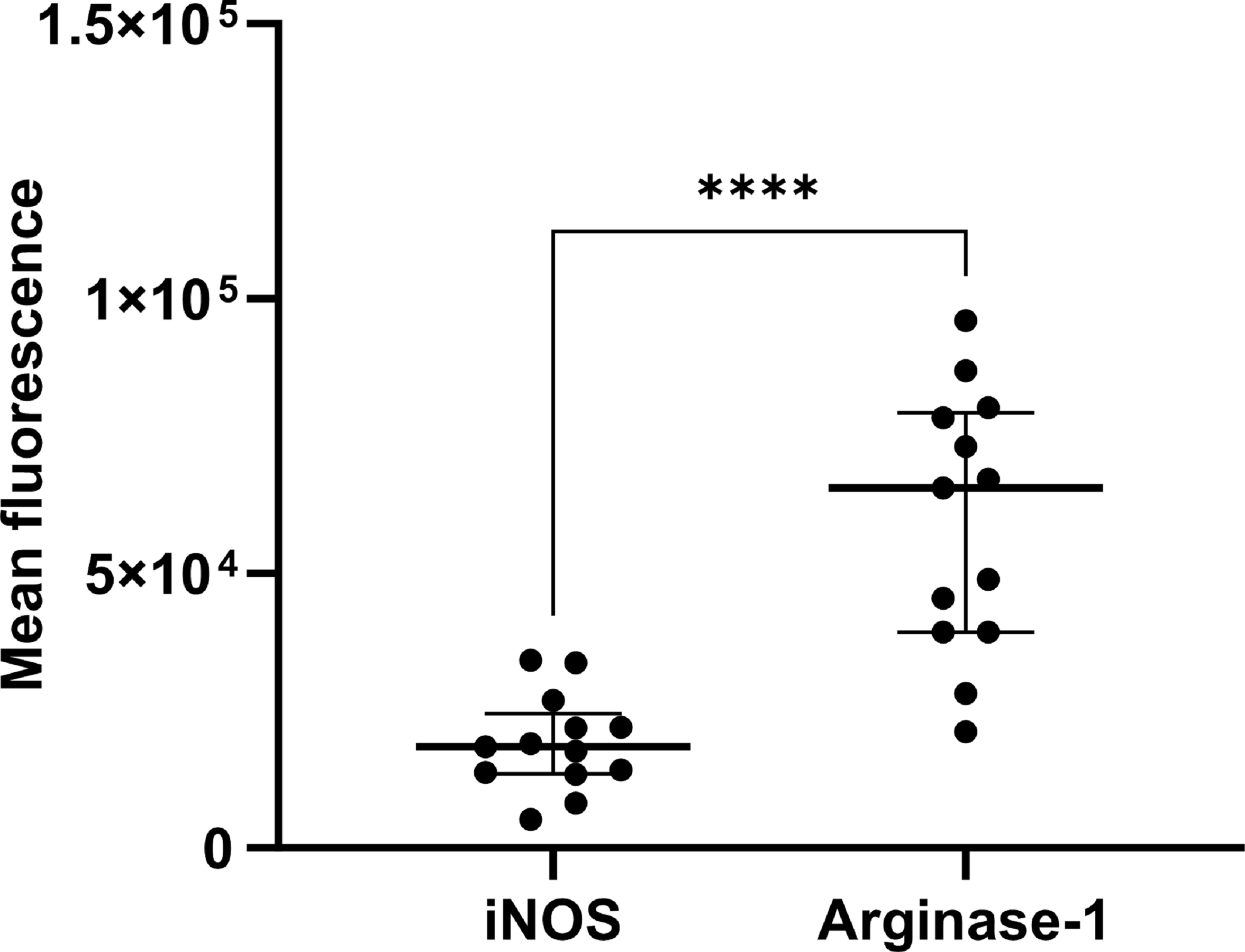

Supplement: Supplementary file 4 — Figure S4: Expression of iNOS and Arginase‐1 enzymes in PBMCs from dogs with leishmaniasis. iNOS and Arginase‐1 expression was assessed in PBMCs from dogs with leishmaniasis (leishmaniasis group, n = 13) and analysed by flow cytometry. Data are expressed as median and interquartile range (25 and 75). Symbols represent individual data for each animal. Unpaired t‐test was performed for group comparisons (****p < 0.0001). [file PIM-48-e70062-s001.tif]

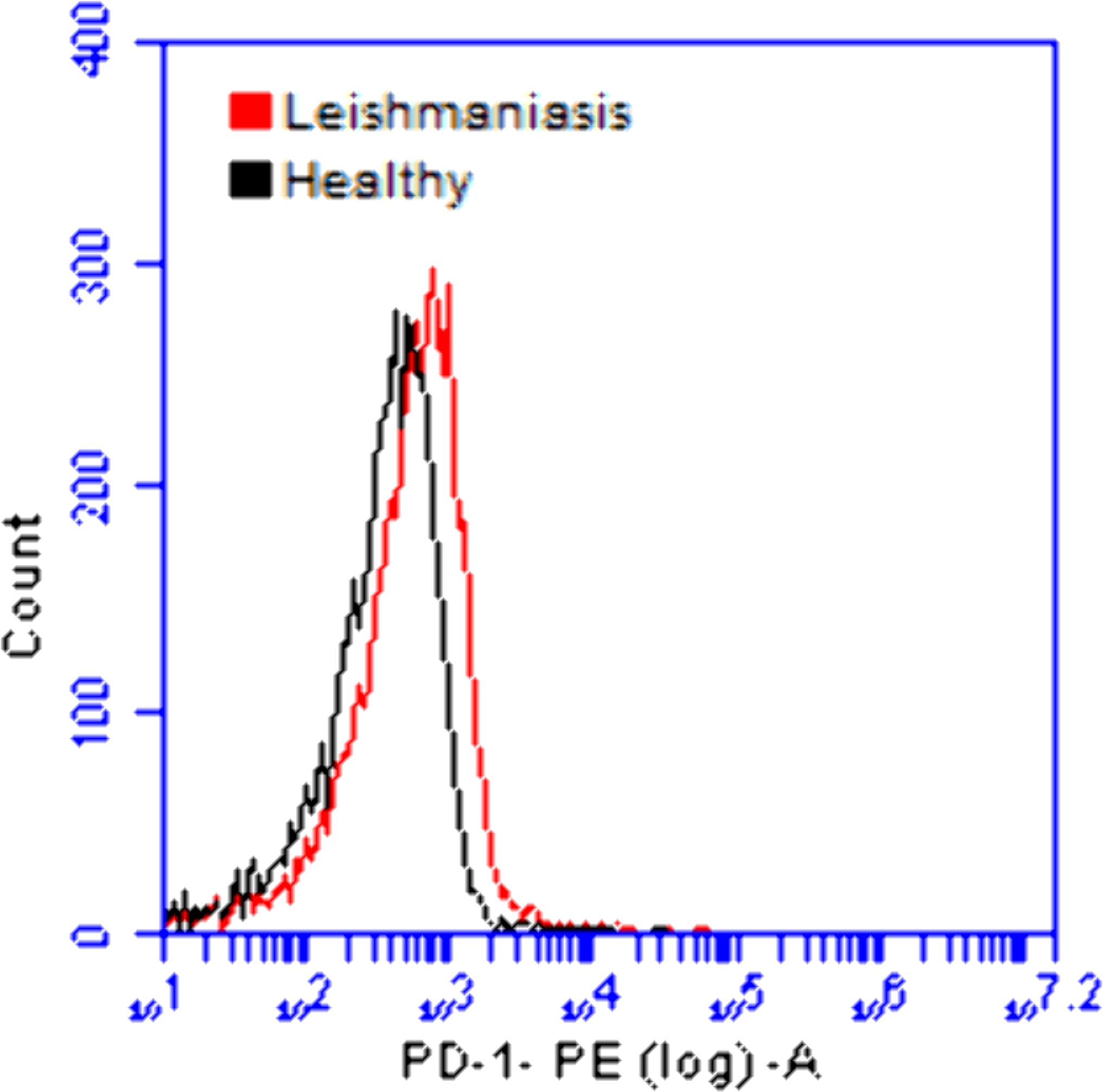

Supplement: Supplementary file 5 — Figure S5: Histogram representing flow cytometry analysis of PD‐1 expression labelling in dogs. PD‐1 expression in PBMCs from dogs was evaluated by flow cytometry and analysed from Gate R2. [file PIM-48-e70062-s004.tif]

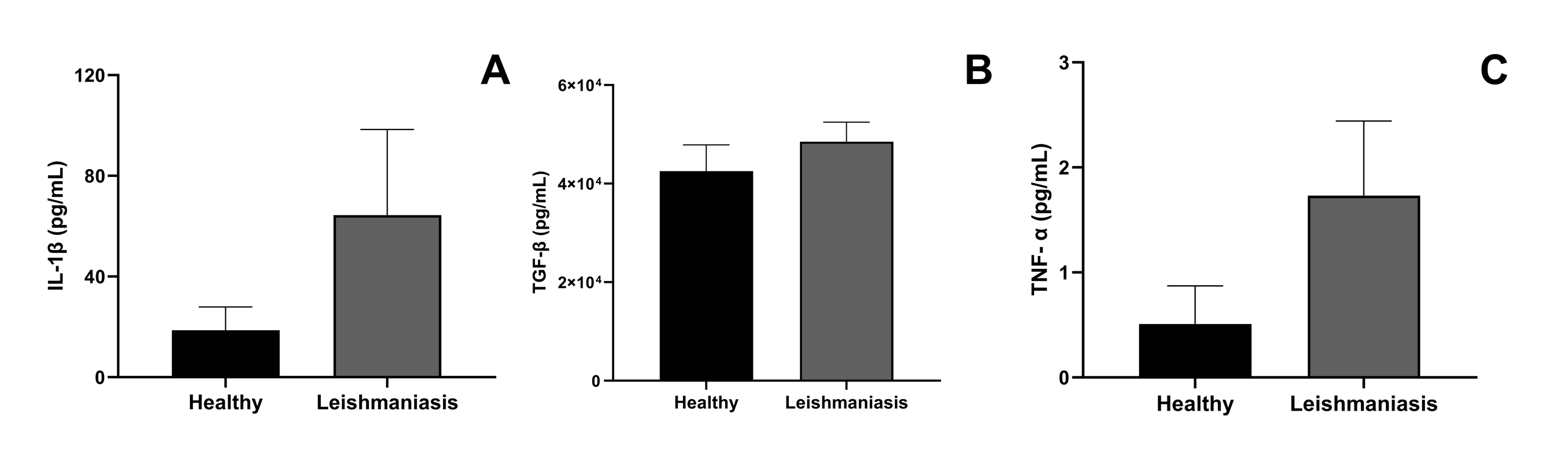

Supplement: Supplementary file 6 — Figure S6: Serum levels of IL‐1β, TGF‐beta and TNF‐α in healthy dogs, dogs with leishmaniasis. Serum levels of cytokines IL‐1β (A), TGF‐beta (B), and TNF‐α were assessed using the capture ELISA method with a commercial kit in healthy dogs (healthy group, n = 10) and dogs with leishmaniasis (leishmaniasis group, n = 13). The data are expressed in bars indicating the mean cytokine levels with standard error of the mean in each group. Unpaired t‐test was performed for group comparisons. Asterisks indicate significant differences (p < 0.05). [file PIM-48-e70062-s007.tif]
