# Supplementary material for: Cortisol Regulates PD‐1 and IL‐12 in Canine Leishmaniasis
Source: Parasite Immunol. 2026 Feb 2;48(2):e70062. doi: 10.1111/pim.70062 (PMC12862539; doi:10.1111/pim.70062)
Supplement: Supplementary file 7 — Table S1: Clinical signs, serological and molecular diagnoses for canine leishmaniasis, and molecular diagnosis of hemoparasitosis in dogs with leishmaniasis (Leishmaniasis) and healthy dogs (Healthy). Table S2: Red blood cells count in dogs with leishmaniasis (Leishmaniasis) and healthy dogs (Healthy). Table S3: White blood cell and platelet count in dogs with leishmaniasis (Leishmaniasis) and healthy dogs (Healthy). Table S4: Biochemical parameters of dogs with leishmaniasis (Leishmaniasis) and healthy dogs (Healthy). Table S5: Correlations between serum cortisol levels, immunological markers, and parasite load in dogs with leishmaniasis. [file PIM-48-e70062-s006.docx]

# Supporting Information

**Figure S1. RFLP analysis of ITS1-PCR amplified fragments from DNA samples of standard isolates using the Hae III enzyme.** M: Molecular marker (100 bp to 2000 bp); LI: *Leishmania infantum*. The samples from dogs with leishmaniasis were identical to those of the *L. infantum* sample. RFLPs were identified on 2% agarose gels stained with red gel, as indicated by an arrow.

**Figure S2. Representative dot plot for gate strategy for selecting monocytes (A) and lymphocytes (B) from a dog with leishmaniasis.** Flow cytometry analyses were performed to assess the fluorescence intensity of iNOS, Arginase-1, and PD-1 on PBMCs from dogs with leishmaniasis and healthy dogs. Monocytes (A) and lymphocytes (B) were separated by size and granularity to form gates R1 and R2, respectively. Representative dot plots from a dog with leishmaniasis. Dots represent individual cells. Gates selected in red represent the percentage of monocytes and lymphocytes. FSC-A (forward scatter) and SSC-A (side scatter).

**Figure S3. Representative histogram of flow cytometry analysis of iNOS and Arginase 1 production in dogs.** iNOS (A) and Arginase-1 (B) production in PBMCs from dogs were evaluated by flow cytometry and analyzed from Gate R1.

**Figure S4. Expression of iNOS and Arginase-1 enzymes in PBMCs from dogs with leishmaniasis.** iNOS and Arginase-1 expression was assessed in PBMCs from dogs with leishmaniasis (leishmaniasis group, n=13) and analyzed by flow cytometry. Data are expressed as median and interquartile range (25 and 75). Symbols represent individual data for each animal. Unpaired t test was performed for group comparisons (****p < 0.0001).

**Figure S5. Histogram representing flow cytometry analysis of PD-1 expression labeling in dogs.** PD-1 expression in PBMCs from dogs was evaluated by flow cytometry and analyzed from Gate R2.

**Figure S6: Serum levels of IL-1β, IL-6, and TNF-α in healthy dogs, dogs with leishmaniasis.** Serum levels of cytokines IL-1β (A), IL-6 (B), and TNF-α were assessed using the capture ELISA method with a commercial kit in healthy dogs (healthy group, n=10) and dogs with leishmaniasis (leishmaniasis group, n=13). The data are expressed in bars indicating the mean cytokine levels with standard error of the mean in each group. Unpaired T Test was performed for group comparisons. Asterisks indicate significant differences (p < 0.05).

**Table S1.** Clinical signs, serological and molecular diagnoses for canine leishmaniasis, and molecular diagnosis of hemoparasitosis in dogs with leishmaniasis (Leishmaniasis) and healthy dogs (Healthy).

| **Dogs** | **Clinical Signs** | **Leishmaniasis Diagnosis** | | | | **Hemoparasitic Infections** | |
| --- | --- | --- | --- | --- | --- | --- | --- |
|  |  | **DPP** | **O.D. ELISA** | **qPCR (Blood)** | **qPCR (Swab)** | **qPCR Ehrlichiosis** | **qPCR Babesiosis** |
| **Leishmaniasis** |  |  |  |  |  |  |  |
| Infected 1 | Lymphadenopathy, onychogryphosis, skin lesions and alopecia | + | 0.754 | + | + | - | - |
| Infected 2 | Lymphadenopathy, onychogryphosis, cachexia, skin lesions, periocular lesions, alopecia, hepatosplenomegaly, and ophthalmic condition | + | 0.579 | + | + | - | - |
| Infected 3 | Lymphadenopathy, onychogryphosis, skin lesions, periocular lesions, and seborrhea | + | 0.887 | - | + | - | - |
| Infected 4 | Lymphadenopathy, onychogryphosis, periocular lesion, and hepatosplenomegaly | - | 0.305 | - | + | - | - |
| Infected 5 | Lymphadenopathy, onychogryphosis, skin lesions, and periocular lesions | - | 0.375 | + | - | - | - |
| Infected 6 | Lymphadenopathy, onychogryphosis, cachexia, periocular lesion, and ophthalmic condition | - | 0.317 | + | - | - | - |
| Infected 7 | Lymphadenopathy, onychogryphosis, skin lesions and alopecia | + | 0.865 | + | - | - | - |
| Infected 8 | Lymphadenopathy, onychogryphosis, skin lesions, and periocular lesions | - | 0.294 | + | + | - | - |
| Infected 9 | Onychogryphosis, skin lesions, periocular lesions, ophthalmic condition, and hepatosplenomegaly | + | 0.580 | + | + | - | - |
| Infected 10 | Onychogryphosis, cachexia, skin lesions, seborrhea and alopecia | + | 0.650 | + | - | - | - |
| Infected 11 | Lymphadenopathy, onychogryphosis, skin lesions, alopecia and hepatosplenomegaly | + | 0.657 | + | + | - | - |
| Infected 12 | Lymphadenopathy, onychogryphosis, skin lesions, periocular lesions, seborrhea, alopecia, ophthalmic condition, and hepatosplenomegaly | + | 0.850 | + | - | - | - |
| Infected 13 | Lymphadenopathy, onychogryphosis, skin lesions, periocular lesions, seborrhea, and hepatosplenomegaly | + | 0.600 | + | + | - | - |
| **Healthy** |  |  |  |  |  |  |  |
| Control 1 | No clinical signs | - | 0.106 | - | - | - | - |
| Control 2 | No clinical signs | - | 0.102 | - | - | - | - |
| Control 3 | No clinical signs | - | 0.210 | - | - | - | - |
| Control 4 | No clinical signs | - | 0.207 | - | - | - | - |
| Control 5 | No clinical signs | - | 0.171 | - | - | - | - |
| Control 6 | No clinical signs | - | 0.092 | - | - | - | - |
| Control 7 | No clinical signs | - | 0.097 | - | - | - | - |
| Control 8 | No clinical signs | - | 0.121 | - | - | - | - |
| Control 9 | No clinical signs | - | 0.115 | - | - | - | - |
| Control 10 | No clinical signs | - | 0.186 | - | - | - | - |
| Control 11 | No clinical signs | - | 0.248 | - | - | - | - |
| Control 12 | No clinical signs | - | 0.150 | - | - | - | - |

Abbreviation: immunochromatographic test (DPP), Polymerase Chain Reaction (PCR), optical density (O.D.). Cut-off point O.D.>0.270

**Table S2.** Red blood cells count in dogs with leishmaniasis (Leishmaniasis) and healthy dogs (Healthy).

| **Dogs** |  | **Red Cells** | **Hemoglobin** | **Hematocrit** | **MCV** | **MCHC** |
| --- | --- | --- | --- | --- | --- | --- |
|  | **Values Reference** | **5.5 - 8.5 x10¹²/mm³** | **12.0-18.0 g/dl** | **37-55 %** | **60-77 fL** | **31-35%** |
| **Leishmaniasis** |  |  |  |  |  |  |
| Infected 1 |  | 5.0 | 9.6 | 29.0 | 57.7 | 33.1 |
| Infected 2 |  | 7.4 | 14.8 | 45.0 | 60.8 | 32.9 |
| Infected 3 |  | 6.4 | 12.8 | 41.0 | 64.1 | 31.2 |
| Infected 4 |  | 7.4 | 17.8 | 52.0 | 70.0 | 34.2 |
| Infected 5 |  | 6.0 | 14.7 | 42.0 | 70.5 | 35.0 |
| Infected 6 |  | 7.8 | 17.3 | 50.0 | 64.1 | 34.6 |
| Infected 7 |  | 3.2 | 8.5 | 24.0 | 74.8 | 35.4 |
| Infected 8 |  | 6.7 | 15.7 | 47.0 | 70.4 | 33.4 |
| Infected 9 |  | 5.2 | 9.3 | 31.0 | 59.5 | 30.0 |
| Infected 10 |  | 3.4 | 7.4 | 24.0 | 70.8 | 30.8 |
| Infected 11 |  | 5.1 | 11.3 | 33.0 | 64.6 | 34.2 |
| Infected 12 |  | 3.5 | 7.2 | 22.0 | 63.0 | 32.7 |
| Infected 13 |  | 4.1 | 8.8 | 26.0 | 63.1 | 33.8 |
|  | Mean± SD | 5.4±1.6^a^ | 11.9±3.8^a^ | 35.8±10.7^a^ | 65.6±5.1^a^ | 33.2±1.7^a^ |
| **Healthy** |  |  |  |  |  |  |
| Control 1 |  | 7.5 | 17.4 | 49.0 | 65.6 | 35.5 |
| Control 2 |  | 7.8 | 18.0 | 54.0 | 69.5 | 33.3 |
| Control 3 |  | 7.7 | 19.3 | 55.0 | 71.2 | 35.1 |
| Control 4 |  | 8.0 | 17.9 | 53.0 | 66.2 | 33.8 |
| Control 5 |  | 7.6 | 17.8 | 51.0 | 67.5 | 34.9 |
| Control 6 |  | 8.5 | 18.6 | 57.0 | 67.3 | 32.6 |
| Control 7 |  | 6.4 | 16.3 | 47.0 | 73.6 | 34.7 |
| Control 8 |  | 7.7 | 18.4 | 53.0 | 68.7 | 34.7 |
| Control 9 |  | 6.3 | 14.2 | 42.0 | 66.9 | 33.8 |
| Control 10 |  | 6.8 | 15.8 | 50.0 | 73.6 | 31.6 |
| Control 11 |  | 6.7 | 15.8 | 46.0 | 68.8 | 34.4 |
| Control 12 |  | 6.7 | 15.2 | 45.0 | 67.3 | 33.8 |
|  | Mean±SD | 7.3±0.7^a^ | 17.1±1.6^a^ | 50.2±4.5^a^ | 68.8±2.7^c^ | 34.0±1.1^c^ |

Abbreviation: mean corpuscular volume (MCV). mean corpuscular hemoglobin concentration (MCHC). standard deviation (SD). Identical letters in identical columns indicate statistical difference using the Unpaired T Test (p < 0.05).

**Table S3.** White blood cell and platelet count in dogs with leishmaniasis (Leishmaniasis) and healthy dogs (Healthy).

| **Dogs** |  | **Neutrophils** | **Lymphocytes** | **Monocytes** | **Eosinophils** | **Platelets** |
| --- | --- | --- | --- | --- | --- | --- |
|  | **Values Reference** | **3.000-11.500 x10^6^/L** | **1.000-4.800 x10^6^/L** | **150-1.350 x10^6^/L** | **150-1.250 x10^6^/L** | **160-440 x10³** |
| **Leishmaniasis** |  |  |  |  |  |  |
| Infected 1 |  | 3.355 | 1.198 | 360 | 1.078 | 206 |
| Infected 2 |  | 8.503 | 761 | 1.650 | 1.777 | 220 |
| Infected 3 |  | 6.052 | 1.958 | 178 | 712 | 204 |
| Infected 4 |  | 3.861 | 660 | 102 | 457 | 229 |
| Infected 5 |  | 3.590 | 1.300 | 248 | 1.052 | 275 |
| Infected 6 |  | 3.660 | 2.379 | 61 | 0 | 200 |
| Infected 7 |  | 2.178 | 1.089 | 0 | 33 | 100 |
| Infected 8 |  | 6.237 | 1.782 | 535 | 356 | 151 |
| Infected 9 |  | 6.358 | 225 | 224 | 598 | 191 |
| Infected 10 |  | 5.143 | 612 | 245 | 122 | 425 |
| Infected 11 |  | 3.995 | 611 | 47 | 47 | 140 |
| Infected 12 |  | 3.902 | 447 | 74 | 74 | 82 |
| Infected 13 |  | 4.818 | 5.735 | 344 | 344 | 140 |
|  | Mean ± SD | 4.742±1.681^a^ | 1.443±1.438^a^ | 312.9±429^a^ | 511.5±529.7^a^ | 197.2±87.1^a^ |
| **Healthy** |  |  |  |  |  |  |
| Control 1 |  | 7.839 | 3.042 | 117 | 702 | 240 |
| Control 2 |  | 6.438 | 2.001 | 174 | 142 | 250 |
| Control 3 |  | 4.697 | 1.098 | 150 | 305 | 240 |
| Control 4 |  | 9.120 | 4.016 | 608 | 456 | 200 |
| Control 5 |  | 8.400 | 1.904 | 448 | 448 | 200 |
| Control 6 |  | 5.200 | 2.640 | 80 | 180 | 240 |
| Control 7 |  | 6.362 | 2.386 | 298 | 894 | 389 |
| Control 8 |  | 3.876 | 2.720 | 136 | 168 | 180 |
| Control 9 |  | 6.144 | 2.372 | 216 | 922 | 236 |
| Control 10 |  | 3.620 | 2.391 | 273 | 346 | 220 |
| Control 11 |  | 3.362 | 3.514 | 382 | 382 | 153 |
| Control 12 |  | 4.421 | 3.039 | 276 | 1.174 | 229 |
|  | Mean ± SD | 5.790±1.923^c^ | 2.594±767.8^a^ | 263.2±154.5^c^ | 509.9±336.9^c^ | 231.4±57.4^b^ |

Abbreviations: standard deviation (SD). Identical letters in identical columns indicate statistical differences. Unpaired t test (neutrophil variable) and Mann-Whitney (for the rest) (p < 0.05).

**S4 Table.** Biochemical parameters of dogs with leishmaniasis (Leishmaniasis) and healthy dogs (Healthy).

| **Dogs** |  | **Albumin** | **ALT** | **AST** | **Creatinine** | **ALP** | **Globulin** | **T. Protein** | **Urea** |
| --- | --- | --- | --- | --- | --- | --- | --- | --- | --- |
|  | **References Values** | **2.6-3.3 g/L** | **21-102 U/L** | **23-66 U/L** | **0.5-1.5 mg/dL** | **20-156 U/L** | **2.7-4.4 g/L** | **5.4-7.1 g/L** | **10.03-50.03 mg/dL** |
| **Leishmaniasis** |  |  |  |  |  |  |  |  |  |
| Infected 1 |  | 2.47 | 26 | 47 | 1.1 | 66 | 7.0 | 9.5 | 25.0 |
| Infected 2 |  | 2.09 | 36 | 41 | 1.0 | 41 | 6.4 | 8.5 | 26.0 |
| Infected 3 |  | 1.80 | 36 | 23 | 1.4 | 24 | 9.0 | 10.8 | 36.0 |
| Infected 4 |  | 3.06 | 41 | 26 | 1.1 | 66 | 4.9 | 8,0 | 27,0 |
| Infected 5 |  | 2.90 | 26 | 26 | 1.1 | 24 | 4.9 | 7.8 | 33.0 |
| Infected 6 |  | 2.36 | 36 | 20 | 0.9 | 74 | 6.2 | 8.6 | 45.0 |
| Infected 7 |  | 2.70 | 32 | 55 | 0.8 | 87 | 7.3 | 10.0 | 26.0 |
| Infected 8 |  | 2.69 | 57 | 26 | 0.9 | 41 | 5.7 | 8.4 | 25.0 |
| Infected 9 |  | 1.41 | 20 | 47 | 1.1 | 182 | 7.8 | 9.2 | 33.0 |
| Infected 10 |  | 1.77 | 31 | 36 | 1.1 | 58 | 5.2 | 7,0 | 47.0 |
| Infected 11 |  | 2.20 | 21 | 88 | 1.0 | 107 | 7.2 | 9.4 | 60.0 |
| Infected 12 |  | 1.11 | 62 | 94 | 0.9 | 265 | 7.8 | 8.9 | 43.0 |
| Infected 13 |  | 1.18 | 57 | 41 | 0.9 | 74 | 6.7 | 7.9 | 19.0 |
|  | Mean ± SD | 2.1±0.6^a^ | 37±13.8^a^ | 43.8±23.5^a^ | 1.0±0.1^a^ | 85.3±67.9^a^ | 6.6±1.2^a^ | 8.8±1.0^a^ | 34.2±11.6^a^ |
| **Healthy** |  |  |  |  |  |  |  |  |  |
| Control 1 |  | 4,0 | 24 | 66 | 1.2 | 23 | 3.6 | 7.6 | 48.1 |
| Control 2 |  | 4,0 | 53 | 61 | 0.8 | 49 | 3,0 | 7,0 | 40.6 |
| Control 3 |  | 3.8 | 41 | 55 | 1.3 | 91 | 4,0 | 7.8 | 29.7 |
| Control 4 |  | 3.3 | 24 | 23 | 1.3 | 154 | 2.9 | 6.2 | 35.0 |
| Control 5 |  | 3.8 | 31 | 61 | 1.2 | 23 | 2.4 | 6.2 | 32.0 |
| Control 6 |  | 4,0 | 30 | 32 | 0.9 | 39 | 3.6 | 7.6 | 24.2 |
| Control 7 |  | 3.03 | 52 | 47 | 1.2 | 41 | 3.37 | 6.4 | 40.0 |
| Control 8 |  | 3.9 | 68 | 28 | 1.1 | 20 | 2.3 | 6.2 | 32.4 |
| Control 9 |  | 2.82 | 41 | 31 | 1.4 | 74 | 2.38 | 5.2 | 29.0 |
| Control 10 |  | 3.05 | 26 | 20 | 1.2 | 49 | 3.45 | 6.5 | 42.0 |
| Control 11 |  | 3.38 | 57 | 31 | 0.7 | 41 | 4,0 | 7.4 | 20.0 |
| Control 12 |  | 3.14 | 57 | 36 | 1.4 | 74 | 2.76 | 5.9 | 47.0 |
|  | Mean ± SD | 3.5±0.4^a^ | 42±15.1^c^ | 40.9±16.2^c^ | 1.2±0.2^c^ | 56.5±37.9^c^ | 3.2±0.6^a^ | 6.8±0.8^a^ | 35±8.8^c^ |

Abbreviation: reference (Ref.). alanine aminotransferase (ALT). aspartate aminotransferase (AST). alkaline phosphatase (ALP). standard deviation (SD). Identical letters in identical columns indicate statistical difference using unpaired t test (albumin, creatinine, globulin, T protein, and urea) and Mann-Whitney test (remaining) (p<0.05).

**Table S5.** Correlations between serum cortisol levels, immunological markers, and parasite load in dogs with leishmaniasis.

| **Variables** | **Cortisol level’s** | |
| --- | --- | --- |
|  | **r** | **p** |
| iNOS PBMC | -0.414 | 0.159 |
| Arginase PBMC | -0.492 | 0.088 |
| IL-1β (pg/ml) | 0.141 | 0.651 |
| IL-6 (pg/ml) | -0.251 | 0.405 |
| IL-10 (pg/ml) | -0.286 | 0.339 |
| IFN-γ (pg/ml) | -0.481 | 0.098 |
| TNF-α (pg/ml) | -0.430 | 0.144 |
| TGF-β (pg/ml) | 0.478 | 0.101 |
| Parasite burden blood | 0.202 | 0.548 |

Abbreviation: inducible nitric oxide synthase (iNOS) peripheral blood mononuclear cells (PBMC). interleukin (IL). picogram (pg). milliliter (ml). interferon (IFN). tumor necrosis factor (TNF). Variables in bold indicate a significant correlation

a. Correlation using Pearson's test for parametric distributions and Spearman's test for nonparametric distributions with correlation coefficients represented by values of *r;*

b. Significance value p<0.05
